# Root-Associated Bacterial Community Shifts in Hydroponic Lettuce Cultured with Urine-Derived Fertilizer

**DOI:** 10.3390/microorganisms9061326

**Published:** 2021-06-18

**Authors:** Thijs Van Gerrewey, Christophe El-Nakhel, Stefania De Pascale, Jolien De Paepe, Peter Clauwaert, Frederiek-Maarten Kerckhof, Nico Boon, Danny Geelen

**Affiliations:** 1 Horticell Lab, Department of Plants and Crops, Ghent University, Coupure Links 653, B-9000 Gent, Belgium; thijs.vangerrewey@ugent.be (T.V.G.); christophe.elnakhel@unina.it (C.E.-N.); 2 Center for Microbial Ecology and Technology (CMET), Ghent University, Coupure Links 653, B-9000 Gent, Belgium; jolien.depaepe@ugent.be (J.D.P.); peterclauwaert@gmail.com (P.C.); frederiekmaarten.kerckhof@ugent.be (F.-M.K.); nico.boon@ugent.be (N.B.); 3 Department of Agricultural Sciences, University of Naples Federico II, 80055 Portici, Italy; depascal@unina.it

**Keywords:** waste streams, source-separated urine, urine-derived fertilizer, organic fertilizer, nutrient cycling, soilless culture, plant holobiont, microbial community, rhizosphere, PGPR

## Abstract

Recovery of nutrients from source-separated urine can truncate our dependency on synthetic fertilizers, contributing to more sustainable food production. Urine-derived fertilizers have been successfully applied in soilless cultures. However, little is known about the adaptation of the plant to the nutrient environment. This study investigated the impact of urine-derived fertilizers on plant performance and the root-associated bacterial community of hydroponically grown lettuce (*Lactuca sativa* L.). Shoot biomass, chlorophyll, phenolic, antioxidant, and mineral content were associated with shifts in the root-associated bacterial community structures. K-struvite, a high-performing urine-derived fertilizer, supported root-associated bacterial communities that overlapped most strongly with control NPK fertilizer. Contrarily, lettuce performed poorly with electrodialysis (ED) concentrate and hydrolyzed urine and hosted distinct root-associated bacterial communities. Comparing the identified operational taxonomic units (OTU) across the fertilizer conditions revealed strong correlations between specific bacterial genera and the plant physiological characteristics, salinity, and NO_3_^−^/NH_4_^+^ ratio. The root-associated bacterial community networks of K-struvite and NPK control fertilized plants displayed fewer nodes and node edges, suggesting that good plant growth performance does not require highly complex ecological interactions in hydroponic growth conditions.

## 1. Introduction

Up to half of the world’s food supply depends on the input of mineral fertilizers [1]. Moreover, a growing population will increase our dependency on fertilizer inputs. Synthetic fertilizer production is scrutinized for its use of non-renewable resources. Phosphorus and potassium fertilizers are mined. The depletion of geological materials and spiking prices jeopardizes the long-term security of P and K [2,3]. The production of N fertilizers starts with the Haber–Bosch synthesis of ammonia. This process requires hydrogen (H_2_), which is mainly derived from natural gas and is responsible for approximately 1.2% of global energy use [4]. In addition, anthropogenically driven deposition of P and N in aquatic and terrestrial ecosystems is the main driver for eutrophication [5]. 

Transitioning to a closed-loop nutrient cycling approach can sustainably provide long-term food security [6]. Human excreta are the primary source of essential nutrients in domestic wastewater. Urine contains an estimated 50% of P and 80% of N present in domestic wastewater while accounting for less than 1% of wastewater volume [7]. Source-separation of urine for fertilizer production can reduce the environmental impact compared to synthetic fertilizers [7,8]. Hilton et al. [7] assessed that fertilizer production from source-separated urine could reduce greenhouse gas emissions by 47%, energy consumption by 41%, eutrophication potential by 64%, and freshwater use by 50% compared to synthetic fertilizer use.

Different techniques exist to recover nutrients from urine [9]. For example, K-struvite precipitation allows for the recovery of K and P [10]. The use of electrodialysis (ED) combined with precipitation and nitrification can recover NPK [11]. Through spontaneous biological hydrolysis of urea, N can be recovered as ammonia or ammonium [12]. The use of urine-derived fertilizers can be as effective as synthetic fertilizers in supporting plant growth [13,14,15,16,17]. However, the application of urine-derived fertilizers in soilless culture systems has received little attention. El-Nakhel et al. [18] explored the use of urine-derived fertilizers in a soilless culture of *Lactuca sativa* L. The application of K-struvite and ED-concentrate nutrient solutions resulted in similar growth performance compared to commercial mineral fertilizer. In addition, K, Ca, Mg, and organic acids accumulated in the lettuce leaves, which can have dietary benefits [18,19]. In contrast, high NH_4_^+^ levels in the hydrolyzed urine nutrient solution resulted in poor growth performance and dark green leaves [18,20,21]. In addition, the high salt content of urine-derived fertilizers can be disadvantageous in soilless systems, especially for a salt-sensitive crop like lettuce [18,22,23].

The root-associated microbial community may be a key factor impacting the effectiveness of urine-derived fertilizers. In the plant holobiont, the complex array of interactions between the plant host and microbes allows them to develop mutually beneficial traits [24,25]. Dynamics between rhizosphere microbes mediate nutrient cycling, potentially influencing plant growth [25,26]. For example, N-fixing microbes, like nodule forming rhizobia, can convert atmospheric N_2_ to readily available nitrogen for plant uptake [27]. Organic N is converted by microbes into plant-available ammonium and nitrate, though plants can also take up simple organic N compounds like amino acids [28,29]. In addition, certain microbial taxa can solubilize phosphorus and iron, improving their plant availability [30,31]. Plants can shape their rhizosphere microbiome to adapt to the environment’s nutrient status to gain benefit over competitors [26]. Other examples of beneficial interactions between the plant host and rhizosphere microbes are the alleviation of salt stress and the protection against pathogens through induced systemic resistance or direct competition between microbes [32,33,34].

Research on the influence of organic fertilizer on rhizosphere microbial community dynamics in soilless culture is limited [35,36]. In soil, conventional and organic agricultural management practices were shown to differ in rhizosphere microbial community structures, which shifted the rhizosphere N-cycling pathways [37]. The application of microalgal biomass as organic fertilizer in a barley soil pot experiment changed the bacterial and protozoan community structures [38]. In soilless culture, the application of organic fertilizer impacted the functionality and microbial associations in the root zone [35,36]. The soil and soilless culture experiments also identified the plant host as an important driver shaping the root-associated microbial community [35,36,37]. In addition, the type of growing medium used in soilless culture can affect the microbial community structure [39,40].

**Figure 1:**
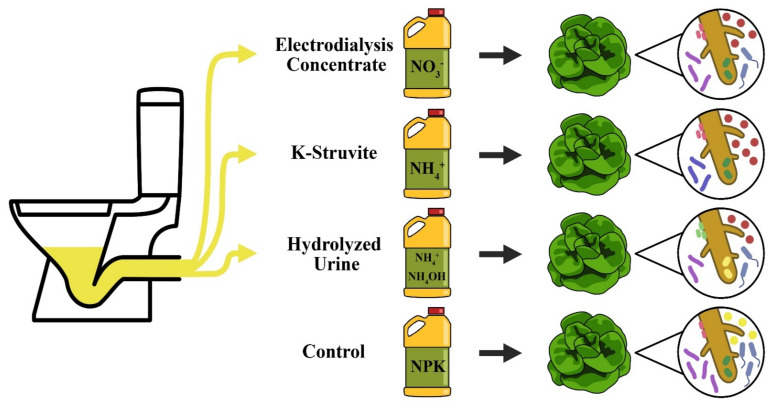
Schematic view of the experiment. Source-separated urine was converted into three urine-derived fertilizers: electrodialysis concentrate, K-struvite, and hydrolyzed urine. These urine-derived fertilizers were applied in a soilless culture of *Lactuca sativa* L. The plant phenotypes, physiological states, and root-associated bacterial communities were evaluated and compared to commercial mineral fertilizer.

The impact of urine-derived fertilizers on the root-associated microbial communities in soilless culture has yet to be elucidated. In this study, we assessed the role of three urine-derived fertilizers (ED concentrate, K-struvite, and hydrolyzed urine) in shaping the root-associated microbial communities of lettuce in soilless culture (Figure 1). We asked the following questions: (1) Does the type of urine-derived fertilizer differently affect lettuce phenotype and physiology? (2) Does the application of urine-derived fertilizers result in distinct root-associated bacterial community structures? (3) Are the key members of the community networks correlated to plant phenotype and physiology as affected by the urine-derived fertilizers’ nutrient status?

## 2. Materials and Methods

### 2.1. Plant Material, Growth Conditions, and Treatments 

A twenty-nine-day plant growth experiment was conducted at the Flanders Research Institute for Agriculture, Fisheries and Food (ILVO), Melle, Belgium (51°0′ N, 3°48′ E) from 12 June until 10 July 2018 (summer season) in a polycarbonate greenhouse. Initially, lettuce seeds (*Lactuca sativa* cv. Grand Rapids TBR, West Coast Seeds, Delta, BC, Canada) germinated on a capillary mat (Aquamat capillary matting, Premier Netting, Norfolk, UK) for 24 h. They then were transferred on Grodan^®^ propagation cubes placed in trays and covered with a dome for the first seven days. After 14 days in a growth chamber, lettuce seedlings were transplanted in 2-liter pots containing 100% Grodan^®^ mini Rockwool Grow-Cubes (Grow Magic Hydroponics, Fulham, London, UK) with a density of 12.5 plants.m^−2^. The maximum daily temperature ranged from 24.4 °C to 35.7 °C, while daily minimum temperature ranged from 18.2 °C to 20.9 °C. Maximum and minimum daily relative humidity ranged from 42.2% to 76.1% and 19.5% to 57.2%, respectively. Lettuce plants were irrigated manually by three different urine-derived fertilizers selected based on lettuce performance in a previous plant growth experiment [18]. The chosen derivatives were the best-performing liquid derivative: ED concentrate, the best-performing solid urine derivate: K-struvite, and the worst-performing derivative: hydrolyzed urine, a liquid derivative. The urine derivatives were prepared as described before [18]. In brief:

1. ED concentrate was prepared by treating human urine with precipitation, nitrification and electrodialysis [11]. NO_3_^−^ was the main N compound;
2. The K-struvite precipitate was produced from human urine by removing all NH_4_-N (below 50 mg N/L), adding an equivalent molar amount of Mg^2+^, and increasing the pH to 10. NH_4_^+^ was the predominant N compound;
3. Hydrolyzed urine was obtained after spontaneous urea hydrolysis during storage of collected human urine at room temperature for several weeks. Total ammonia N (TAN; NH₄OH and NH_4_^+^) was the main N compound.

The final urine-derived nutrient solutions were composed as described by El-Nakhel et al. [18] and were applied manually every other day using a laboratory beaker. An industry-standard NPK 20-10-20 + trace elements (TE) nutrient solution was used as the control treatment. The pH of all the nutrient solutions was adjusted to 6.0 before the application using NaOH or H_2_SO_4_. A randomized complete block design was used in this experiment, with treatments replicated three times. Each experimental unit consisted of 6 plants.

### 2.2. Plant Sample Analysis

#### 2.2.1. Soil Plant Analysis Development Index, Biomass Determination, Growth Analysis, Total N, Mineral Content, and Organic Acids Content Analysis

Plant samples were analyzed as previously described by El-Nakhel et al. [18]. In brief, the soil plant analysis development (SPAD) index was measured right before harvest using a portable chlorophyll meter SPAD-502 (Konica-Minolta, Tokyo, Japan). After harvest, plant biomass and growth were determined by measuring leaf number, leaf area, fresh weight (FW), dry weight (DW), and dry matter content (% DM) of the aerial plant parts. Dried leaf tissues were ground and used for total N, mineral (NO_3_^−^, NH_4_^+^, PO_4_^3−^, SO_4_^2−^, Cl^−^, K^+^, Ca^2+^, Mg^2+^, and Na^+^), and organic acids (malate, tartrate, oxalate, citrate, and isocitrate) content analysis. The total N concentration was determined by the Kjeldahl method [41]. The minerals and organic acids were separated and quantified by ion chromatography (ICS-3000, Dionex, Sunnyvale, CA, USA) coupled to a conductivity detector using an IonPac CG12A guard column and IonPac analytical column for cations and an IonPac AG11-HC guard column and IonPac AS11-HC analytical column for anions and organic acids [42]. All minerals and organic acids were expressed as g.kg^−1^ DW, except for isocitrate expressed as mg.kg^−1^ DW. As for NO_3_^−^, it was expressed as mg.kg^−1^ FW, based on each sample dry matter percentage.

#### 2.2.2. Chlorophylls and Carotenoids Content Analysis

Chlorophyll a (Chl_a_), chlorophyll b (Chl_b_), and carotenoids were determined according to Lichtenthaler and Buschmann [43]. The photosynthetic pigments were quantified after an 80% acetone extraction of the fresh sample. The absorbance of the extracts was read at 470 nm, 646.8 nm, and 663.2 nm wavelengths. The amount of Chl_a_, Chl_b_, and carotenoids (C_x+c_) was calculated in μg.mL^−1^ according to the following equations:

Chl_a_ = 12.25 × A_663.2_ − 2.79 × A_646.8_ (1)

Chl_b_ = 21.5 × A_646.8_ − 5.1 × A_663.2_ (2)

C_x+c_ = (1000 × A_470_ − 1.82 × Chl_a_ − 85.02 × Chl_b_)/198 (3)

#### 2.2.3. Total Phenolic Content

Total phenolic content (TPC) was evaluated using the Folin–Ciocalteu method [44], with gallic acid (Sigma Aldrich Inc., St Louis, MO, USA) as a standard. In brief, 10 mL of 80% methanol was added to 250 mg of fresh sample, tubes were placed in a sonicator at room temperature for 30 min, and centrifuged for 5 min at 2000× *g*. The supernatant (20 μL) was combined with 100 μL of Folin–Ciocalteau’s reagent (Sigma Aldrich Inc., St Louis, MO, USA), and after 4 min, 80 μL of sodium carbonate (75 g.L^−1^) was added. After 2 h of incubation in darkness at room temperature, the absorbance was measured at 765 nm (InfiniteM200, TECAN Group Ltd., Switzerland). TPC was expressed as µg gallic acid equivalents (GAE) per 100 g FW.

#### 2.2.4. Total Antioxidant Capacity

Total antioxidant capacity (TAC) was analyzed according to Re et al. [45] with L-ascorbic acid (L-AA) as a standard. This assay involves the oxidation of ABTS (2,2′-azinobis[3-ethylbenzothiazoline-6-sulphonate]) to an intensely-colored N-centered radical cation, ABTS·. The intensely colored ABTS· radical is obtained by adding 1 mL 20 mM ABTS to the working solution (7 mL 75 mM KH_2_PO_4_, 1 mL 1.75% H_2_O_2_, and 1 mL 0.08% horseradish peroxidase). Fresh leaf material (300 mg) was extracted with 600 μL extraction buffer (3% metaphosphoric acid, 1 mM EDTA, and 2% PVPP), followed by centrifugation at 5600× *g* 4 °C. The resulting pellet was extracted again with an extraction buffer, and both supernatants were pooled. The aliquot (20 µL) was added to the 180 μL ABTS working solution, and after 6 min, the remaining ABTS· was quantified spectrophotometrically at 734 nm (InfiniteM200, TECAN Group Ltd., Switzerland). TAC was expressed as μmol L-AA equivalents per 100 g FW.

#### 2.2.5. Plant Sample Statistical Analysis

Group means from all data were analyzed using analysis of variance (ANOVA) via the SPSS software package (v. 20.0). Duncan’s multiple range test (DMRT) was performed for mean comparisons on each of the significant (*p* < 0.05) variables measured.

### 2.3. Root-Associated Bacterial Community Analysis

#### 2.3.1. Root-Associated Bacterial Community Sample Collection

Root-associated bacterial samples were collected after harvest following the procedure described by Barillot et al. [46] and Van Gerrewey et al. [40] with slight modifications. In brief, for each treatment, three plant root systems (1 plant root system per treatment replicate) were pooled together, and 3 g of roots were sampled. Furthermore, three separate plant roots were sampled from a single ED-treated plant to confirm intra-treatment homogeneity. The sampling of the roots was repeated four times. The root samples were processed further to isolate three root zones: the rhizosphere, the rhizoplane, and the combined rhizoplane/endosphere.

Rhizosphere bacterial communities were collected by adding the root samples to 50 mL of a sterile 0.9% NaCl washing solution. The samples were subsequently placed on a rotary shaker at 150 × rpm for 90 min. The roots were removed and placed in a sterile container. After, the washing solution was centrifuged at 4300× *g* for 15 min. The resulting pellet was vortexed and centrifuged again at 4300× *g* for 15 min. Finally, the supernatant was discarded, and the pellet was stored at −80 °C. For identifying the combined rhizoplane/endosphere bacterial communities, 200 mg of the washed roots set aside in a sterile container during the rhizosphere sample processing were sampled and frozen at −80 °C before freeze-drying. For the rhizoplane bacterial community isolation only, the remaining washed roots that were set aside were rewashed in sterile 0.9% NaCl + 0.01% Tween 80 and shook on a rotary shaker for 90 min at 150 × rpm, and then pelleted and frozen following the same method used for the rhizosphere sampling.

Samples of the four different nutrient solution treatments were taken before application. Each sample was centrifuged for 15 min, at 4300× *g*, twice. Each time the supernatant was discarded. The final remaining pellet was frozen at −80 °C before being freeze-dried for further analyses.

#### 2.3.2. 16S rRNA Gene Amplicon Sequencing

16S rRNA gene amplicon sequencing was performed as described before [47,48]. In brief, DNA extraction was performed using the Zymo Research Zymobiomics DNA kit (Irvine, CA, USA), following the manufacturer’s instructions. The 16S rRNA gene V3-V4 hypervariable regions were amplified by PCR using primers Bakt_341F (5′- CCT ACG GGN GGC WGC AG-3′, pB-3844) and Bakt_805R (5′- GAC TAC HVG GGT ATC TAA TCC-3′, pB-3845). The reverse primer was adapted from Klindworth et al. [49] to increase coverage. PCR was performed using *Taq* DNA Polymerase with the Fermentas PCR Kit according to the manufacturers’ specifications (Thermo Fisher Scientific, Waltham, MA, USA). The obtained PCR product was run on a 2% agarose gel for 30 min at 100 V. Ten μL of the original genomic DNA extract was sent out to BaseClear B.V. (Leiden, The Netherlands) for library preparation and sequencing on an Illumina Miseq platform with v3 chemistry and the primers mentioned above. For assessing the sequencing quality, a mock community was included in triplicate in the sequencing run. Read assembly and cleanup were derived mainly from the MiSeq SOP described by the Schloss lab [50,51]. In brief, mothur (v. 1.44.3) was used to remove primers and barcodes, assemble reads into contigs, perform alignment-based quality filtering (alignment to the mothur-reconstructed SILVA SEED alignment, v. 138), remove chimeras (vsearch v. 2.13.3), and assign taxonomy using a naïve Bayesian classifier [52] and SILVA NR v. 138 [53]. These contigs were clustered into operational taxonomic units (OTU) at 97% sequence similarity. All sequences that were classified as Eukaryota, Archaea, Chloroplasts, and Mitochondria were removed. If sequences could not be classified at all (even at (super) Kingdom level), they were removed. For each OTU, the representative sequence was picked as the most abundant sequence within that OTU cluster. The raw fastq files used to create the OTU table have been deposited in the National Center for Biotechnology Information (NCBI) database (accession number PRJNA726879).

#### 2.3.3. Root-Associated Bacterial Community Statistical Analysis

All statistical analyses were performed in R (v. 4.0.3) using the phyloseq package (v. 1.34.0) to import the data [54,55]. Singleton OTUs were considered noise and were removed from the dataset before analysis. After removing singleton OTUs, 611,033 16S rRNA gene sequences from 48 root samples remained in the data set with an average of 12,730 sequences per sample (Figure S1). The 16S rRNA gene sequences represented 2636 OTUs, which consisted of 22 phyla, 45 classes, 111 orders, 183 families, and 380 genera.

Three alpha diversity metrics were estimated, taking both unobserved rare taxa and measurement error into account using the method described by Willis et al. [56]. When sampling a microbial population from a natural environment, the entire population is rarely obtained, resulting in rare taxa missing from samples [56]. However, the estimation of alpha diversity indices is heavily dependent on sample size. Samples with a more extensive library size are more likely to detect more taxa, resulting in an increased alpha diversity estimation [57]. Rarefaction is a commonly used method to address the issues that result from differences in sampling depth [58]. Though rarefaction of count data is considered invalid when comparing relative abundances [59].

Moreover, technical replicates in microbiome experiments produce different results means that there is a measurement error [57,60]. Therefore, to compare the actual total alpha diversity of a microbiome, it is required to consider both the taxa missing from the samples and the uncertainty resulting from using samples as proxies for the entire microbiome. The breakaway package (v. 4.7.1) was used to estimate richness (i.e., the total number of taxa) by fitting a nonlinear regression model to the ratios of consecutive frequency counts allowing the estimation of the unobserved taxa [61]. The DivNet package (v. 0.3.7) was used to estimate Shannon’s diversity and Simpson’s diversity at the genus level, taking unknown taxa into account [62]. Both the Shannon and Simpson diversity indices allow us to quantify alpha diversity accounting for both the richness and evenness (i.e., the distribution of taxa relative abundances) of the lettuce root-associated bacterial samples. Shannon’s diversity index describes the uncertainty of predicting individual taxa, which becomes more difficult with an increasing number of taxa and equal taxon relative abundance [63]. Simpson’s diversity index corresponds to the probability that two random observations taken from the sample represent the same OTU [64]. After quantifying the alpha diversity indices, the breakaway package betta_random() function statistically compared the alpha diversity indices between urine-derived fertilizer treatments or root zones using the variance of the estimates in a mixed model approach [56]. When fitting the model with urine-derived fertilizer treatment as a fixed effect, the root zone was added as a random effect and vice versa.

Beta-diversity was analyzed by Principal Coordinates Analysis (PCoA) based on the abundance-based Bray–Curtis dissimilarity matrix (phyloseq package) and visualized with the ggplot2 package (v. 3.3.3) [65]. A PerMANOVA (permutations = 10,000; α = 0.05) on the Bray–Curtis distances evaluated the homogeneity of variances and determined differences in community structure between the urine-derived fertilizer treatments and the root zones with Holm adjustment for multiple comparisons (vegan package v. 2.5-7) [66].

Differential abundance testing was used to identify abundant bacteria in the urine-derived fertilizer treatments compared to the NPK control group and associate bacteria with high or low plant performance. The differential abundance tests were performed with the DESeq2 package (v. 1.30.1) using a Wald test with a local fit (α = 0.01) [67].

Indicator taxon analysis using multi-level pattern analysis (permutations = 10,000) with the indicator value index “IndVal.g” of Dufrêne and Legendre [68] was utilized to identify indicator OTUs for each treatment and root zone (indicspecies package v. 1.7.9) [69]. *p*-values were adjusted for multiple testing with the Benjamini–Hochberg adjustment.

Before constructing microbial networks for each treatment and root zone, the OTU table was filtered by keeping only OTUs present in more than 40% of the samples using the seqtime package (v. 0.1.1) to reduce sparsity [70,71]. Furthermore, a dummy row containing the sum of the filtered out OTUs was added to the filtered OTU table not to change the total sample count. The SpiecEasi package (v. 1.1.0) was used to normalize the filtered data and compute the sparse inverse covariance matrix using the Meinshausen–Buhlmann neighborhood selection method [72]. The Stability Approach to Regularization Selection (StARS) was used to estimate the optimal penalty parameter λ, with a minimal λ ratio set to 5e-5, the number of λ penalties set to 100, and the number of subsamples for StARS set to 20. The resulting adjacency matrix was then converted into an igraph object, removing unconnected nodes. The igraph package (v. 1.2.6) was used to visualize the microbial networks and detect network clusters (using the fast greedy modularity optimization algorithm) and hubs (using Kleinberg’s hub centrality scores) [73].

## 3. Results

### 3.1. Does the Type of Urine-Derived Fertilizer Differently Affect Lettuce Phenotype and Physiology?

The effectiveness of the urine-derived fertilizers ED concentrate, hydrolyzed urine, and K-struvite was compared to NPK (20-10-20 + TE) fertilizer by cultivating lettuce seedlings in 2-liter pots containing mini Rockwool cubes. After 29 days, the growth performance was determined by measuring leaf number, leaf area, FW, DW, and % DM (Table S1). The performance of lettuce grown with K-struvite was similar to the NPK control fertilized plants, in contrast to plants grown with ED concentrate and hydrolyzed urine. The ED concentrate and hydrolyzed urine fertilized lettuce were smaller, generated fewer leaves, and exhibited lower FW and DW values. The % DW was higher than in NPK control and K-struvite, indicating a significant change in metabolism. Overall, the hydrolyzed urine fertilized lettuce showed the poorest performance, significantly lower than ED concentrate grown plants.

The substantial difference in performance prompted us to assess the stress levels and compare the lettuce quality. Photosynthetic pigments (SPAD, chlorophylls, and carotenoids), stress metabolites (TAC and TPC), and organic acids (malate, tartrate, oxalate, citrate, and isocitrate) were determined (Tables S2 and S3). The photosynthetic pigment concentrations were similar in NPK control and K-struvite fertilized plants. The hydrolyzed urine fertilized plants showed the highest concentration in photosynthetic pigments. The photosynthetic pigment concentrations were the lowest in lettuce treated with ED concentrate fertilizer. Treatment with ED concentrate fertilizer resulted in the highest accumulation of stress metabolites and organic acids. The stress metabolite concentrations of hydrolyzed urine fertilized plants were similar to NPK control and K-struvite, while the organic acid concentrations were the lowest amongst all treatments.

As urine is rich in nitrogen (N), total N, NO_3_^−^, and NH_4_^+^ content in the plants were analyzed (Table S4). The total N in K-struvite and NPK control-treated plants was similar, which presumably contributed to vigorous growth on these fertilizers. In K-struvite, the highest accumulation of NO_3_^−^ was recorded (4463 mg.kg^−1^ FW), despite that NH_4_^+^ was the primary source of N. The total N in lettuce treated with hydrolyzed urine was very high (5.35% DW). The high NH_4_^+^ content (1.26 mg.kg^−1^ DW) in the hydrolyzed urine lettuce was the main contributor to total N, as NO_3_^−^ content (44 mg.kg^−1^ FW) was more than 70-fold lower than NPK control (3162 mg.kg^−1^ FW). TAN was the primary source of N in this treatment, which coincides with the high NH_4_^+^ and low NO_3_^−^ levels. Plants treated with ED concentrate contained the lowest total N (3.60% DW), as NO_3_^−^ content was 2160 mg.kg^−1^ FW, but NH_4_^+^ content (0.45 mg.kg^−1^ DW) was the lowest among the fertilizer treatments.

Other mineral nutrients (PO_4_^3-^, K^+^, Ca^2+^, Mg^2+^, Na^+^, Cl^−^, and SO_4_^2−^) were also analyzed (Table S4). Fertilization with urine-derived fertilizer deviated from the NPK control plants by the accumulation of Na^+^ and Cl^−^. In particular Na^+^ accumulated excessively upon ED concentrate (31.05 g.kg^−1^ DW) and K-struvite treatment (14.09 g.kg^−1^ DW), whereas Cl^−^ accumulated in plants treated with ED concentrate (17.55 g.kg^−1^ DW) and hydrolyzed urine (37.63 g.kg^−1^ DW). These results coincide with the high concentrations of NaCl in urine, the dosage of NaOH during the precipitation and nitrification steps of the ED concentrate preparation, and the dosage of NaOH during the pH adjustment of the K-struvite nutrient solution. K-struvite treated plants contained a higher dose of Mg^2+^ (6.42 g.kg^−1^ DW) than NPK control (3.87 g.kg^−1^ DW), which coincides with the addition of Mg^2+^ during the preparation of the K-struvite precipitate. SO_4_^2−^ concentrations were also higher in lettuce fertilized with hydrolyzed urine (11.72 g.kg^−1^ DW) and K-struvite (9.35 g.kg^−1^ DW) compared to NPK control (3.26 g.kg^−1^ DW).

### 3.2. Does the Application of Urine-Derived Fertilizers Result in Distinct Root-Associated Bacterial Community Structures?

**Figure 2:**
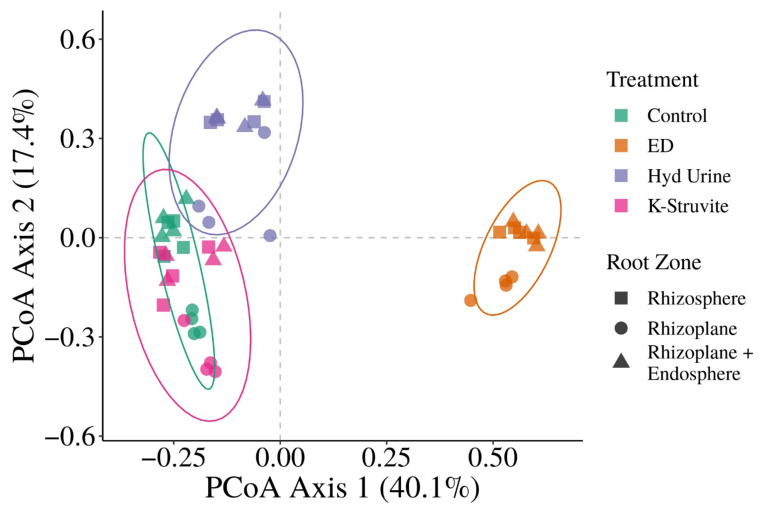
Bray-Curtis dissimilarity Principle Coordinates Analysis (PCoA) of the lettuce root-associated bacterial community samples. Colors indicate the urine-derived fertilizer treatment (ED: electrodialysis concentrate, Hyd Urine: hydrolyzed urine), and symbols indicate the root zone of the samples (4 replicates). Ellipses are drawn using a multivariate t-distribution.

Visualization of the Bray–Curtis dissimilarity matrix using Principle Coordinates Analysis (PCoA) revealed great differences in bacterial community structure between the urine-derived fertilizer treatments (Figure 2). The first and second axes (PCoA axis 1 and PCoA axis 2) explained 40.1% and 17.4% of the Bray–Curtis dissimilarity matrix variation. The ED concentrate samples clustered far apart from the other urine-derived fertilizer treatments along the first axis. NPK control and K-struvite, the highest yielding fertilizers, clustered together and separated from the hydrolyzed urine treatment. The root zones showed a clustering of the rhizosphere and the combined rhizoplane/endosphere samples while separating from the rhizoplane along the second axis. A PerMANOVA on the Bray-Curtis distances showed significant differences in community structure explained by the urine-derived fertilizer treatments (R^2^ = 0.56; *p* < 0.0001) and the root zones (R^2^ = 0.15; *p* = 0.0006) (Tables S5 and S6). Pairwise comparison between treatment groups showed significant differences for all comparisons. However, testing for homogeneity of variances between the urine-derived fertilizer treatments revealed that the K-struvite and NPK control treatment groups’ variances were significantly different from each other (*p* = 0.0396). Thus, the observed PerMANOVA significant pairwise comparison between K-struvite and NPK control likely stems from a difference in variance rather than a difference in community structure. Pairwise comparison of the root zones indicated significant differences in community structure between the rhizosphere and the rhizoplane (R^2^ = 0.13; *p* = 0.006), and between the rhizoplane and combined rhizoplane/endosphere (R^2^ = 0.15; *p* = 0.006). The community structure did not significantly differ between the rhizosphere and the combined rhizoplane/endosphere (R^2^ = 0.07; *p* = 0.063).

**Figure 3:**
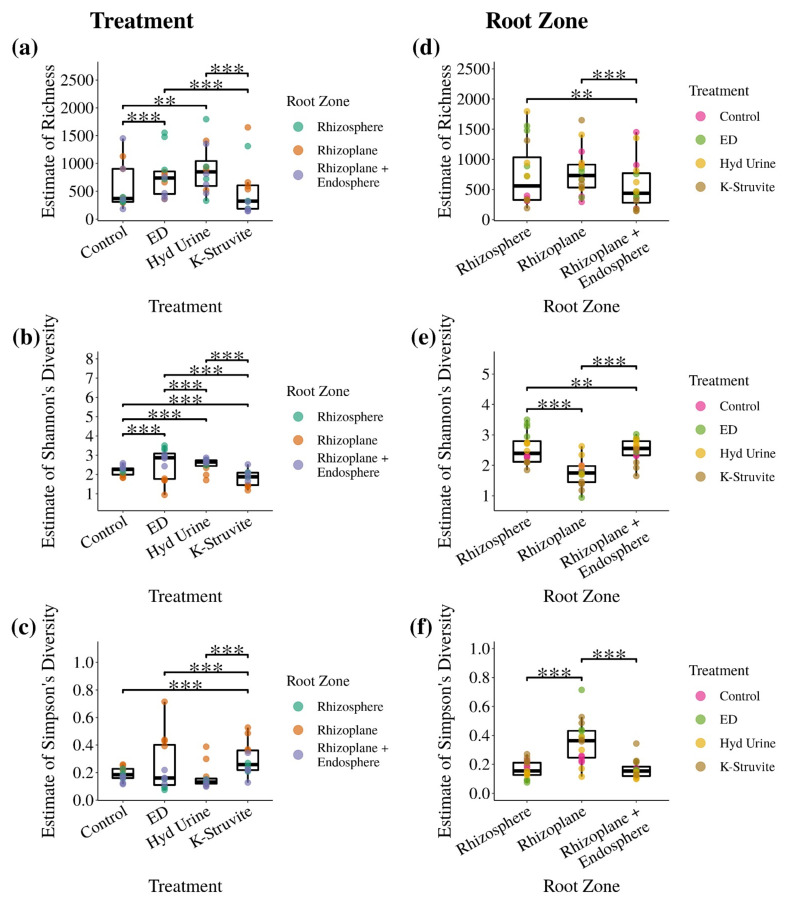
Boxplots of the richness, Shannon’s diversity, and Simpson’s alpha diversity indices of the lettuce root-associated bacterial communities grouped per urine-derived fertilizer treatment (ED: electrodialysis concentrate, Hyd Urine: hydrolyzed urine) (**a**–**c**) or root zone (**d**–**f**). The alpha diversity indices were estimated by taking unknown taxa into account. Shannon’s diversity and Simpson’s diversity indices were determined at the genus level. Statistical comparison of the alpha diversity indices between urine-derived fertilizer treatments or root zones using the estimates’ variance in a mixed model approach. When fitting the model with urine-derived fertilizer treatment as a fixed effect, the root zone was added as a random effect and vice versa. Only significant pairwise comparisons are shown. Asterisks indicate level of significance: *p* < 0.01 (**) and *p* < 0.001 (***). N per urine-derived fertilizer treatment = 12 and *n* per root zone = 16.

The impact of the urine-derived fertilizers on root-associated bacterial community diversity was assessed by estimating three alpha diversity metrics: bacterial richness, Shannon’s diversity index, and Simpson’s diversity index (Figure 3). Plants fertilized with hydrolyzed urine showed a significantly higher estimated richness (i.e., the total number of OTUs) by 312 ± 111 OTUs (mean ± SE) compared to NPK control (*p* = 0.005) (Figure 3a). Similarly, the estimated total richness in the ED treatment was significantly higher by 164 ± 45 OTUs than the NPK control (*p* < 0.001). K-struvite fertilization showed a significant reduction in richness by 226 ± 40 OTUs and 374 ± 41 OTUs compared to the ED (*p* < 0.001) and hydrolyzed urine (*p* < 0.001) treatments, respectively. However, no difference in estimated root-associated bacterial richness was observed between the K-struvite and NPK control treatments (*p* = 0.132). The hydrolyzed urine and the ED concentrate treatments did not differ in richness (*p* = 0.166). Shannon’s diversity (i.e., the uncertainty of predicting individual taxa) at the genus level was significantly higher in the ED concentrate treatment (*p* < 0.001) and the hydrolyzed urine treatment (*p* < 0.001) compared to the NPK control (Figure 3b). Lettuce root-associated bacterial samples from the ED concentrate treatment had the highest Shannon diversity index of the three urine-derived fertilizers (*p* < 0.001). The K-struvite treatment resulted in the lowest Shannon diversity (*p* < 0.001). Since Simpson’s diversity index calculates the probability that two random observations taken from a sample represent the same OTU, this means that a high Simpson’s diversity index coincides with a low alpha diversity and vice versa. The K-struvite Simpson diversity index at the genus level was the highest (*p* < 0.001) (Figure 3c). Simpson’s diversity index did not significantly differ between the NPK control, the ED concentrate, and the hydrolyzed urine treatments. Overall, the three alpha diversity metrics indicate a higher root-associated bacterial community diversity in the inferior performing ED concentrate and hydrolyzed urine treatments compared to the well-performing K-struvite and NPK control treatments.

Comparing the diversity metrics between the root zones (Figure 3d–f) revealed significant effects of the root sampling location on alpha diversity. The combined rhizoplane/endosphere root zone significantly decreased by 115 ± 36 OTUs on average compared to the rhizosphere (*p* = 0.002) and by 170 ± 34 OTUs compared to the rhizoplane (*p* < 0.001). The lettuce rhizosphere richness did not significantly differ from the rhizoplane richness (*p* = 0.169), and the rhizoplane had the lowest Shannon diversity index (*p* < 0.001). The combined rhizoplane/endosphere had the highest Shannon diversity index on average, significantly higher than the rhizosphere (*p* = 0.001). Simpson’s diversity index was the highest in the rhizoplane samples (*p* < 0.001) but did not significantly differ between the rhizosphere and rhizoplane/endosphere root zones (*p* = 0.297). Overall, the three diversity metrics indicated that though the number of genera in the rhizoplane was high, a few selected genera dominated this root zone. In contrast, the number of genera in the combined rhizoplane/endosphere was low, but they were evenly abundant.

Estimating the OTU log_2_ fold changes identified multiple differentially abundant OTUs between the urine-derived fertilizers and NPK control. ED concentrate and NPK control differed in 222 OTUs, with 167 OTUs more abundant in the ED treatment and 55 OTUs more abundant in the control (Figure S2). A total of 83 OTUs were differentially abundant between hydrolyzed urine and NPK control, with 61 OTUs being more abundant in the hydrolyzed urine treatment and 22 OTUs being more abundant in the NPK control treatment (Figure S3). When comparing OTU log_2_ fold changes between K-struvite and NPK control, 29 OTUs were differentially abundant between both treatment groups (Figure S4). Of the 29 OTUs, eight OTUs were more abundant in the K-struvite treatment, and 21 OTUs were more abundant in the NPK control.

To determine which bacterial OTUs best defined the response to urine-derived fertilizer, indicator analysis was performed, identifying 14 indicator OTUs in the NPK control (Table S7). *Pseudomonas* was the only genus in the NPK control with an indicator value above 0.90 (0.99). The *Burkholderiaceae* and *Sphingomonadaceae* families were most represented with 3 OTUs each, followed by *Pseudomonadaceae* and *Enterobacteriaceae* with 2 OTUs, and the remaining families represented by a single OTU only. A total of 185 indicator OTUs were identified in the ED concentrate treatment group (Table S8). Nine genera had a maximal indicator value: *Devosia*, *Gemmatimonas*, *Leptospira*, *Aminobacter*, *SH-PL14*, *Sphingomonas*, *Arcicella*, *Flectobacillus*, and *Pseudoxanthomonas*. The *Burkholderiaceae* family was most represented with 29 OTUs, followed by *Rhizobiaceae* (17 OTUs), *Chitinophagaceae* (11 OTUs), *Pseudomonadaceae* (9 OTUs), *Flavobacteriaceae* (9 OTUs), *Spirosomaceae* (9 OTUs), *Sphingobacteriaceae* (6 OTUs), *Sphingomonadaceae* (6 OTUs), and *Xanthobacteraceae* (5 OTUs). Less than 5 OTUs represented the remaining families. The hydrolyzed urine treatment was represented by 36 indicator OTUs (Table S9). There were six genera with an indicator value of 0.90 or higher: an unclassified genus from the *Burkholderiaceae* family (0.99), *Edaphobacter* (0.97), an uncultured genus from the *Micropepsaceae* family (0.95), *Eoetvoesia* (0.92), *Zoogloea* (0.91), and *JGI_0000069-P22_ge* (0.90). The *Burkholderiaceae* family, with 11 indicator OTUs, was the only family containing more than five indicator OTUs. Nine indicator OTUs were identified in the K-struvite urine-derived fertilizer treatment (Table S10). Only an unclassified *Enterobacteriaceae* (0.98) and *Thiomonas* (0.96) had an indicator value above 0.90. The *Enterobacteriaceae* family was most represented with 3 OTUs, followed by *Burkholderiaceae* and *Rhodanobacteraceae* with 2 OTUs each, and the remaining families represented by a single OTU.

Overall, the indicator taxa analysis showed that specific genera represent the urine-derived fertilizer treatments, confirming that the treatments induce the formation of distinct root-associated bacterial communities. The shift in bacterial structure compared to the NPK control is especially prevalent in the poor performing ED concentrate and hydrolyzed urine-derived fertilizers, with many more differentially abundant OTUs. In contrast, few differentially abundant OTUs were identified in the K-struvite treatment, which performed equally to NPK control. Indicator taxa analysis did not identify any root zone-specific indicator OTUs and few differentially abundant OTUs were detected between the root zones (Figures S5–S7), in agreement with an overlap in bacterial structure between the root zones.

### 3.3. Are the Key Members of the Community Networks Correlated to Plant Phenotype and Physiology as Affected by the Urine-Derived Fertilizers’ Nutrient Status?

**Figure 4:**
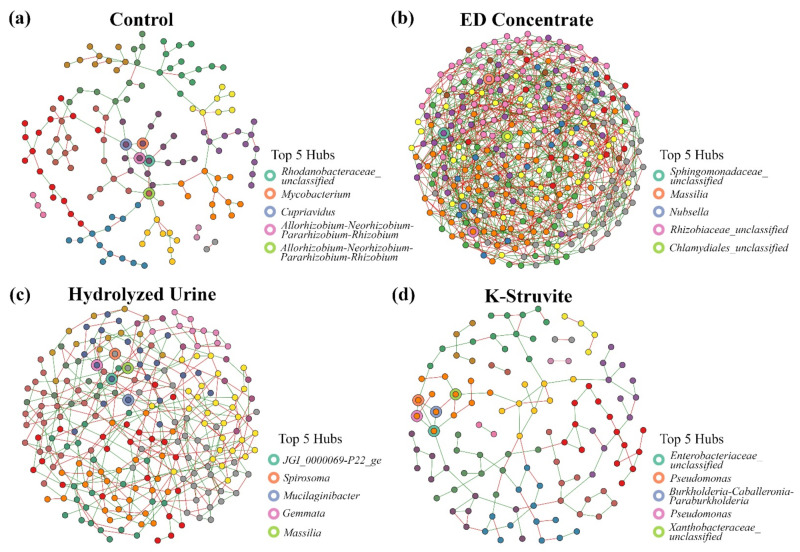
Lettuce root-associated bacterial community networks grouped per urine-derived fertilizer treatment: (**a**) NPK control network with 141 nodes, 149 edges (102 positives and 47 negatives), and 15 clusters with a 0.82 modularity (i.e., a measure for how good the division in clusters is); (**b**) Electrodialysis (ED) concentrate network with 311 nodes, 964 edges (539 positives and 425 negatives), and 9 clusters (modularity = 0.45); (**c**) Hydrolyzed urine network with 222 nodes, 413 edges (228 positives and 185 negatives), and 11 clusters (modularity = 0.60); (**d**) K-struvite network with 120 nodes, 130 edges (92 positives and 38 negatives), and 15 clusters (modularity = 0.79). Nodes are colored by cluster (clusters were determined using the fast greedy modularity optimization algorithm). The top 5 hub nodes are indicated by the colored borders (hubs were determined using Kleinberg’s hub centrality scores). The green and red edges indicate a positive or negative correlation between the network nodes, respectively.

Sparse inverse covariance matrices were computed to determine the bacterial community structure and correlation level of bacteria across the urine-derived fertilizer treatments. After conversion to adjacency matrices, the root-associated bacterial community networks were visualized in Figure 4. The NPK control and K-struvite treatments showed a lower node count (141 and 120 nodes respectively) than the poor performing ED concentrate and hydrolyzed urine (311 and 222 nodes, respectively). The NPK control and K-struvite also had a lower average amount of edges per node (1.06 and 1.08 edges per node, respectively) compared to ED concentrate and hydrolyzed urine (3.10 and 1.86 edges, respectively). The percentage of positive and negative correlations between the nodes in the ED concentrate and hydrolyzed urine networks were equally divided (approximately 55% positive and 45% negative edges). In contrast, most of the nodes in the NPK control and K-struvite networks were positively correlated (approximately 70% positive and 30% negative edges).

The node degree (i.e., the number of adjacent edges of a node) and hub scores of each node were calculated to identify key genera in the urine-derived fertilizer bacterial community networks. When identifying the highest node degree in each urine-derived fertilizer treatment network (Table S11), three OTUs had a maximal node degree of 11 in the ED concentrate treatment: OTU26 (*Cupriavidus*), OTU185 (an unclassified *Burkholderiaceae*), and OTU618 (an unclassified *Chlamydiales*). OTU57 (*Sphingobium*) and OTU169 (*JGI_0000069-P22_ge*) had a maximal node degree of 8 in the hydrolyzed urine treatment. There was one OTU (OTU85, an unclassified *Rhodanobacteraceae*) with a maximal node degree of 6 in the NPK control treatment. In the K-struvite treatment, four OTUs had a maximal node degree of 5: OTU8 (*Allorhizobium-Neorhizobium-Pararhizobium-Rhizobium*), OTU13 (*Herbaspirillum*), OTU36 (*Sphingomonas*), and OTU473 (an unclassified *Enterobacteriaceae*).

Kleinberg’s hub centrality score was used to detect hubs present in urine-derived fertilizer treatment networks (Table S12). The NPK control network had three genera with a hub score above 0.80: OTU85 (an unclassified *Rhodanobacteraceae*) (1), OTU252 (*Mycobacterium*) (0.81), and OTU26 (*Cupriavidus*) (0.81). The hub scores of two OTUs in the K-struvite network were higher than 0.80: OTU473 (an unclassified *Enterobacteriaceae*) (1) and OTU325 (*Pseudomonas*) (0.83). In the hydrolyzed urine network, the hub scores of both OTU169 (*JGI_0000069-P22_ge*) and OTU393 (*Spirosoma*) were higher than 0.80 (1 and 0.88, respectively). The ED concentrate network contained 15 genera with a hub score above 0.80, of which the hub score of five genera was even higher than 0.90: OTU68 (an unclassified *Sphingomonadaceae*) (1), OTU14 (*Massilia*) (0.98), OTU142 (*Nubsella*) (0.96), OTU499 (an unclassified *Rhizobiaceae*) (0.95), and OTU618 (an unclassified *Chlamydiales*) (0.91).

The rhizosphere revealed a much more complex interaction network with 237 nodes than the rhizoplane with 173 nodes and the combined rhizoplane/endosphere with 145 nodes (Figure S8). The rhizosphere nodes were connected by 2.34 edges per node on average, followed by the rhizoplane (1.74 edges per node) and combined rhizoplane/endosphere (1.63 edges per node). The rhizosphere and rhizoplane showed a similar percentage of positive and negative correlations between nodes (approximately 67% positive and 33% negative edges). The percentage of positive correlations was lower in the combined rhizoplane/endosphere network than the other root zones (60% positive and 40% negative edges).

Identifying the critical genera in the root zone networks based on the number of adjacent edges (node degree) revealed one OTU in the rhizosphere network with the highest node degree of 11 (OTU51, an unclassified *Fimbriimonadaceae*) (Table S13). In the rhizoplane network, four OTUs had a maximal node degree of 9: OTU4 (*Pandoraea*), OTU10 (*Burkholderia-Caballeronia-Paraburkholderia*), OTU109 (*Hyphomicrobium*), and OTU122 (*Sphingobium*). Three OTUs had a maximal node degree of 8 in the rhizoplane/endosphere network: OTU6 (*Pseudomonas*), OTU7 (*Burkholderia-Caballeronia-Paraburkholderia*), and OTU14 (*Massilia*) (Table S13).

Key hub genera were determined in each root zone network (Table S14). The rhizosphere network had two OTUs with a hub score of 0.80 or higher: OTU46 (*Reyranella*) (1) and OTU51 (an unclassified *Fimbriimonadaceae*) (0.80). Two OTUs with a hub score above 0.80 were identified in the rhizoplane network: OTU109 (*Hyphomicrobium*) (1) and OTU10 (*Burkholderia-Caballeronia-Paraburkholderia*) (0.96). The combined rhizoplane/endosphere network contained four OTUs with a more than 0.80 hub score: OTU8 (*Allorhizobium-Neorhizobium-Pararhizobium-Rhizobium*) (1), OTU7 (*Burkholderia-Caballeronia-Paraburkholderia*) (0.95), OTU40 (*Pelomonas*) (0.90), and OTU36 (*Sphingomonas*) (0.86) (Table S14).

The correlations between differentially abundant OTUs and lettuce performance and nutritional composition metrics were determined to assess a putative role for root zone bacteria with lettuce performance and quality (Table S15). Differentially abundant OTUs were identified across all root samples by log_2_ fold change per unit increase of each plant metric. An extra filtering step was implemented to associate the differentially abundant OTUs with a specific urine-derived fertilizer treatment by selecting the more abundant OTUs in the urine-derived fertilizer treatments than NPK control. After filtering, the ED concentrate treatment had 166 OTUs associated with at least one of the plant metrics, followed by hydrolyzed urine (60 OTUs) and K-struvite (8 OTUs).

Key OTUs correlated to the plant metrics were selected based on their log_2_ fold change value, the number of plant metrics they were correlated to, their identification as an indicator OTU, and their network node degree and hub score. ED concentrate fertilized plants showed low growth performance, were stressed, had low N-levels, and accumulated salt. Multiple key OTUs in the ED concentrate root-associated bacterial community were linked to the urine-derived fertilizer’s poor performance. Two OTUs, can unclassified *Caulobacteraceae* (OTU205) and *Kaistia* (OTU127), correlated to poor plant performance, showed a high node degree (a node degree of 10 for both genera) and hub score (ranked 13th and 18th, respectively). Interestingly, three ED concentrate indicator OTUs of the *Pseudomonas* genus (OTU6, OTU24, and OTU80) positively correlated to leaf number while also correlating to high stress levels, low N-levels, and high salt levels. Although ED concentrate fertilized lettuce showed poor growth performance overall, the leaf number did not differ from NPK control. The three Pseudomonads may have played a role in the plants’ leaf development, although overall growth performance was poor. The nine ED concentrate indicator OTUs with a maximal indicator value of 1 were linked to a high stress response, low N-levels, and high salt levels: OTU284 (*Devosia*), OTU139 (*Gemmatimonas*), OTU105 (*Leptospira*), OTU195 (*Aminobacter*), OTU194 (*SH-PL14*), OTU135 (*Sphingomonas*), OTU113 (*Arcicella*), OTU160 (*Flectobacillus*), and OTU112 (*Pseudoxanthomonas*). As well as the indicator OTUs classified as the genera *Aeromonas* (OTU18), *Acidovorax* (OTU27), and *Pedobacter* (OTU39), which showed high log_2_ fold change values. Five more indicator OTUs that correlated to high stress metrics, low N-levels, and high salt levels were also key players in the ED concentrate bacterial network, as they had a high node degree and hub score: OTU142 (*Nubsella*; node degree 9; hub rank 3), OTU303 (*NS9_marine_group_ge*; node degree 9; hub rank 7), OTU67 (*Methylophilus*; node degree 10; hub rank 8), OTU51 (an unclassified *Fimbriimonadaceae*; node degree 10; hub rank 12), and OTU185 (an unclassified *Burkholderiaceae*; node degree 11; hub rank 54).

Hydrolyzed urine fertilized plants had low growth performance, high % DW, high levels of chlorophylls, high TAC, high total N with high NH_4_^+^-N but low NO_3_^—^N levels, high Cl^−^, and high SO_4_^2-^. Multiple significant players in the hydrolyzed urine root-associated bacterial community were connected to the plants’ physiological status. Four indicator OTUs with high node degree and hub score were correlated to all hydrolyzed urine plant metric statuses: OTU169 (*JGI_0000069-P22_ge*; node degree 8; hub rank 1), OTU172 (an uncultured *Holosporaceae*; node degree 7; hub rank 20), OTU421 (*Zoogloea*; node degree 6; hub rank 30), and OTU307 (*Eoetvoesia*; node degree 5; hub rank 32). Three more indicator OTUs strongly correlated to the hydrolyzed urine fertilized lettuce’s physiological status were: OTU213 (*Edaphobacter*), OTU235 (*FBP_ge*), and OTU245 (an unclassified *Acetobacteraceae*). Five essential network OTUs classified as the genera *Massilia* (OTU76 and OTU14; node degrees 6 and 7; hub ranks 5 and 23), *OPB56_ge* (OTU123; node degree 5; hub rank 16), *Sphingobium* (OTU57; node degree 8; hub rank 18), and *Dyadobacter* (OTU33; node degree 5; hub rank 22) also showed strong correlations to the hydrolyzed urine plant metric statuses. Although OTU46 (*Reyranella*) was not identified as an indicator or important network hub, it did show strong correlations to the hydrolyzed urine plants’ poor growth performance. OTUs identified as members of the *Burkholderiaceae* family showed significant log_2_ fold changes in correspondence to the hydrolyzed urine fertilized lettuce’s physiological status. Moreover, *Burkholderiaceae* relative abundance was the highest in the hydrolyzed urine treatment (50.74%) (Figure S9).

K-struvite fertilized lettuce showed similar performance, chlorophylls, and stress metabolite levels to NPK control. Furthermore, levels of NO_3_^−^, NH_4_^+^, Na^+^, Mg^2+^, and SO_4_^2−^ were high. PCoA showed that the root-associated bacterial communities did differ between K-struvite and NPK control. However, differential abundance and indicator OTU analysis revealed a few representative OTUs for the K-struvite treatment. These differentially abundant OTUs showed strong correlations to the K-struvite fertilized lettuce’s physiological status. Two families showed strong correlations with the K-struvite lettuce physiological status: *Rhodanobacteraceae* (OTU56, OTU72, and OTU400) and *Enterobacteriaceae* (OTU180 and OTU361). Two of the three *Rhodanobacteraceae* OTUs (OTU56 and OTU400) and both *Enterobacteriaceae* OTUs were indicator OTUs for the K-struvite root-associated bacterial community. OTU349, classified as genus *Thiomonas*, another K-struvite indicator OTU, also correlated strongly to the plant metric statuses. OTU22 classified as genus *Burkholderia-Caballeronia-Paraburkholderia* with high node degree (4) and hub rank (21st) positively correlated to K-struvite stress metrics, NH_4_^+^, and SO_4_^2−^ levels. Moreover, this OTU was also a key player in the hydrolyzed urine treatment. Both K-struvite and hydrolyzed urine-derived fertilizer had NH_4_^+^ as the primary N compound, indicating that members of the *Burkholderiaceae* family may play a role in the oxidation of NH_4_^+^.

## 4. Discussion

Previous research has shown that urine-derived fertilizers can be applied successfully in a hydroponics environment [18,74,75,76]. However, little is known about how the root-associated bacterial communities are influenced in a hydroponics environment by organic fertilizer application, particularly urine-derived fertilizer [35,36]. Here we show that root-associated bacterial communities are profoundly altered by the type of urine-derived fertilizer applied. Poor performing urine-derived fertilizer stimulated a rich bacterial community, whereas the strong performing K-struvite was linked with a less complex community similar to the NPK control. The urine-derived fertilizers were selected based on their contrasting performance in a previous plant growth experiment [18], where hydrolyzed urine showed poor performance, and K-struvite and ED concentrate showed high performance similar to a commercial mineral fertilizer [18]. Contrary to this previous study [18], the ED concentrate performance was lower than the NPK control. Low photosynthetic pigment concentrations and accumulation of stress metabolites indicated the ED concentrate fertilized plants were stressed. Because the greenhouse temperature was up to 10 °C higher compared to the previous experiment [18], it may have differentially affected the performance of the urine derived fertilizer [77].

Comparing the bacterial diversity between the root zones showed that the rhizoplane had the highest bacterial richness. Although high in the number of OTUs, the rhizoplane was dominated by unclassified *Enterobacteriaceae* and *Pseudomonas*, accounting for more than 60% in OTU relative abundance (Figure S9). In general, the bacterial diversity found in plant root zones is represented mainly by OTUs belonging to the genera *Bacillus*, *Pseudomonas*, *Enterobacter*, *Arthrobacter*, *Rhizobium*, *Agrobacterium*, *Burkholderia*, *Azospirillum*, *Azotobacter*, *Mycobacterium*, *Flavobacterium*, *Cellulomonas,* and *Micrococcus* [78]. Indeed, *Enterobacter*, *Pseudomonas*, *Burkholderia*, *Rhizobium*, and *Azospirillum* were among the top 12 genera present in our lettuce root-associated bacterial community samples (Figure S9), affirming that the plant select bacteria from a core microbiome to generate a distinct bacterial community in function of the fertilizer [79].

Our results further indicate that urine-derived fertilizers differentially shift the root-associated microbiome structure. The log_2_ fold changes identified many differentially abundant OTUs between the ED concentrate and hydrolyzed urine treatments compared to NPK control, while only a few OTUs were differentially abundant between K-struvite and NPK control. Similarly, Grunert et al. [35], Robles-Aguilar et al. [36], and Suleiman et al. [38] observed differences in root bacterial communities due to the application of organic fertilizers. However, they also identified the plant host as a major determinant for bacterial community structure. Multiple studies have demonstrated that the plant host shapes the root microbial community, with plant root exudates acting as signal molecules regulating the interactions between the plant and microbes [79,80,81]. In our study, many OTUs observed in the nutrient solutions were not present in the root environment (Figures S10–S14). The apparent differences in bacterial communities between the nutrient solution and the root zone point to the importance of the plant host in assembling its root-associated bacterial community.

The use of Rockwool, a growing medium typically low in nutrients and low in bacterial counts, may have contributed to limited competition with growing medium-born genera [39,82]. Indeed, Mallon et al. [83] observed an increase in microbial community diversity after invasion, with less diverse communities experiencing significant shifts in niche structure. The fact that only the poor-performing urine-derived fertilizers associated with a profound shift in bacterial community structure might indicate that the weakened plants’ root systems were more susceptible to invasion by a broader range of genera, explaining the more diverse root-associated bacterial communities and more extensive community networks in the poor-performing ED concentrate and hydrolyzed urine treatments compared to K-struvite and NPK control. Moreover, ED concentrate and hydrolyzed urine introduced sodium salts and an unbalanced NO_3_^−^/NH_4_^+^ ratio, resulting in suboptimal plant growth [18,20,84]. In addition, the higher ratio of negative correlations between network OTUs in the ED concentrate and hydrolyzed urine treatments compared to NPK control and K-struvite indicate a stronger competition between bacteria for occupying the community niches.

Most importantly, multiple key genera were linked to the urine-derived fertilizers’ effect on plant phenotype and physiology. Overall, these key genera are commonly found in the rhizosphere of plants and saline or aquatic environments and can use NH_4_^+^ or NO_3_^−^ as an N source, can solubilize phosphate, and show plant growth-promotion or pathogenic properties (Table S16). This indicates that these key genera were well adapted to our experimental hydroponics environment, which gave them a head start in occupying the different niches.

For example, in the poor performing ED concentrate treatment, *Acidovorax* was identified as an indicator genus. *Acidovorax* has been detected in the lettuce rhizosphere [85]. Moreover, members of the *Acidovorax* genus are pathogenic, causing bacterial leaf spots in corn salad and bacterial fruit blotch in watermelon [86,87]. *Aminobacter*, an ED concentrate indicator genus, can form root nodules and use NH_4_^+^, NO_3_^−^, and urea as an N source [88,89]. Another ED concentrate indicator genus, *SH-PL14,* is a member of the *Planctomycetales,* is characterized by anaerobic oxidation of NH_4_^+^ to N_2_ (anammox) [90,91]. *Flectobacillus*, a third ED concentrate indicator genus, has been detected in rice paddy rhizosphere soil and can solubilize phosphate and potassium [92,93,94]. Surprisingly, three OTUs identified as *Pseudomonas* were positively correlated to leaf number in the ED concentrate treatment, suggesting they promoted shoot growth. Salt tolerant Pseudomonads have been shown to improve the growth of several plant species (mustard, sunflower, citrus, soybean) under salt stress conditions [95,96,97,98]. The Pseudomonads that were detected in the ED concentrate fertilized plants likely alleviated salt stress improving shoot growth.

Key genera in the poor performing hydrolyzed urine treatment are plant pathogenic, while others show antifungal activity. *Reyranella* was not identified as an indicator or hub genus but did show strong correlations to the hydrolyzed urine plants’ poor growth performance. *Reyranella* has been detected in a eutrophic lake and the rhizosphere of lettuce [99,100]. Moreover, *Reyranella* may promote the outbreak of bacterial wilt disease (*Ralstonia solanacearum*) [101]. *Sphingobium*, a hydrolyzed urine network hub, is known to cause corky root in lettuce which is induced by free NH_4_^+^ [102,103]. TAN (NH₄OH and NH_4_^+^) was the main N compound in the hydrolyzed urine fertilizer, which may have promoted the establishment of *Sphingobium* in the lettuce rhizosphere. In addition, *Sphingobium* can degrade allelopathic pterostilbene, produced by plants to inhibit fungal infection, limiting its effectiveness [104]. Two hydrolyzed urine network hub genera (*Massilia* and *Dyadobacter*) show antifungal activity. *Massilia* inhibits *Phytophthora infestans* and is linked to *Rhizoctonia solani* suppression [105,106,107]. Moreover, *Massilia* can produce indole-3-acetic acid (IAA; a phytohormone), indicating plant growth-promoting traits [108]. *Dyadobacter* can suppress Fusarium wilt and rice blast disease, and *Dyadobacter* has been detected in seawater, indicating the genus is well adjusted to a saline environment [109,110]. The fact that the key genera *Massilia* and *Dyadobacter* show antifungal activity and are commonly in high abundance in the rhizosphere of infected plants may indicate that a fungal pathogen infected the hydrolyzed urine fertilized plants.

Finally, multiple K-struvite indicator OTUs of the families *Rhodanobacteraceae* (3 OTUs) and *Enterobacteriaceae* (2 OTUs) were positively correlated to increased plant performance. Indeed, both families have known plant growth-promoting properties. Members of the *Rhodanobacteraceae* are endophytes in tomato root, and they have been isolated from NO_3_^−^-rich subsurface environments [111,112]. *Rhodanobacteraceae* members promote plant growth under salt-stressed conditions, produce antioxidant enzymes, are capable of complete denitrification, and show antagonism towards *Fusarium solani* and *Ralstonia solanacearum* [111,112,113]. Members of the *Enterobacteriaceae* also promote plant growth as they can produce phytohormones, solubilize phosphate and fix N [78,114,115]. Members of this family have been shown to inhibit *Fusarium solani* growth, thereby helping the plant host combat fungal pathogens [114].

## 5. Conclusions

Nutrient recovery from urine is a sustainable alternative for mined mineral fertilizers that can be successfully applied in soilless cultures. K-struvite was a high-performing fertilizer in this study and was revealed to support root-associated bacterial communities similar to the control NPK fertilizer. The poor performing ED concentrate and hydrolyzed urine fertilizers showed very different bacterial communities linked to high salinity and imbalances in NO_3_^−^ and NH_4_^+^ ratio. Further research should focus on removing these high levels of NaCl, ensuring a balanced NO_3_^−^/NH_4_^+^ ratio, and investigating whether the bacterial communities can protect the plant from pathogen invasion. In addition, for urine-derived fertilizers to complement and eventually even replace mineral fertilizer, we need to ensure no harmful microbes associate with the cultivated crop.

## Data Availability

The raw fastq files used to create the OTU table have been deposited in the National Center for Biotechnology Information (NCBI) database (accession number PRJNA726879).

## Supplementary Materials

The following are available online at https://www.mdpi.com/article/10.3390/microorganisms9061326/s1, Figure S1: Rarefaction curves of each of the urine-derived fertilizer samples divided by root zone, Figure S2: Differentially abundant OTUs between the ED concentrate and NPK control-treated lettuce root-associated bacterial community samples, Figure S3: Differentially abundant OTUs between the hydrolyzed urine and NPK control-treated lettuce root-associated bacterial community samples, Figure S4: Differentially abundant OTUs between the K-struvite and NPK control-treated lettuce root-associated bacterial community samples, Figure S5: Differentially abundant OTUs between the rhizoplane and rhizosphere lettuce root-associated bacterial community samples, Figure S6: Differentially abundant OTUs between the combined rhizoplane/endosphere and rhizosphere lettuce root-associated bacterial community samples, Figure S7: Differentially abundant OTUs between the combined rhizoplane/endosphere and rhizoplane lettuce root-associated bacterial community samples, Figure S8: Lettuce root-associated bacterial community networks grouped per root zone, Figure S9: Relative abundance of the top 12 genera present in the lettuce root-associated bacterial community samples grouped per urine-derived fertilizer treatment and root zone. Figure S10: Relative abundance of the top 12 genera in the different urine-derived fertilizer’s nutrient solutions before application, Figure S11: Differentially abundant OTUs between the NPK control-treated lettuce root-associated bacterial community samples and the NPK control nutrient solution, Figure S12: Differentially abundant OTUs between the ED concentrate-treated lettuce root-associated bacterial community samples and the ED concentrate nutrient solution, Figure S13: Differentially abundant OTUs between the hydrolyzed urine-treated lettuce root-associated bacterial community samples and the hydrolyzed urine nutrient solution, Figure S14: Differentially abundant OTUs between the K-struvite-treated lettuce root-associated bacterial community samples and the K-struvite nutrient solution, Table S1: Lettuce leaf number, leaf area, fresh weight (FW), dry weight (DW), and dry matter content regarding the urine-derived fertilizer treatments, Table S2: Lettuce leaf photosynthetic pigments and stress metabolites in respect to the urine-derived fertilizer treatments, Table S3: Lettuce organic acid content in respect to the urine-derived fertilizer treatments, Table S4: Lettuce leaf mineral analysis in respect to the urine-derived fertilizer treatments, Table S5: PerMANOVA with Holm adjustment for multiple comparison on Bray-Curtis distances and homogeneity of variances explained by the urine-derived fertilizers, Table S6: PerMANOVA with Holm adjustment for multiple comparison on Bray-Curtis distances and homogeneity of variances explained by root zone, Table S7: Indicator OTUs for the lettuce root-associated bacterial community NPK control samples ordered by the indicator value, Table S8: Top 20 indicator OTUs for the lettuce root-associated bacterial community ED concentrate samples ordered by the indicator value, Table S9: Top 20 indicator OTUs for the lettuce root-associated bacterial community hydrolyzed urine samples ordered by the indicator value, Table S10: Indicator OTUs for the lettuce root-associated bacterial community K-Struvite samples ordered by the indicator value, Table S11: The top 10 genera ordered by node degree in the lettuce root-associated bacterial community networks grouped per urine-derived fertilizer treatment, Table S12: The top 10 hubs ordered by Kleinberg’s hub centrality score in the lettuce root-associated bacterial community networks grouped per urine-derived fertilizer treatment, Table S13: The top 10 genera ordered by node degree in the lettuce root-associated bacterial community networks grouped per root zone, Table S14: The top 10 hubs ordered by Kleinberg’s hub centrality score in the lettuce root-associated bacterial community networks grouped per root zone, Table S15: Differentially abundant OTUs linked to the lettuce performance and nutritional composition metrics grouped per fertilizer treatment, Table S16: Overview of the habitats and characteristics of the key genera and families in the lettuce root-associated bacterial communities fertilized with urine-derived fertilizer. References [116,117,118,119,120,121,122,123,124,125,126,127,128,129,130,131,132,133,134,135,136,137,138,139,140,141,142,143,144,145,146,147,148,149,150,151,152,153,154,155,156,157,158,159,160,161,162,163,164,165,166,167,168,169,170] are cited in the Supplementary Materials.
