# Supplementary material for: Root-Associated Bacterial Community Shifts in Hydroponic Lettuce Cultured with Urine-Derived Fertilizer
Source: Microorganisms. 2021 Jun 18;9(6):1326. doi: 10.3390/microorganisms9061326 (PMC8233860; doi:10.3390/microorganisms9061326)
Supplement: Supplementary file 1 [file microorganisms-09-01326-s001.zip › microorganisms-1255426-supplementary_TVG_20210617/UrineDerivativeLettuceMicrobiome_Supplementary_20210617_TVG.pdf]

Supplementary Material

Figures

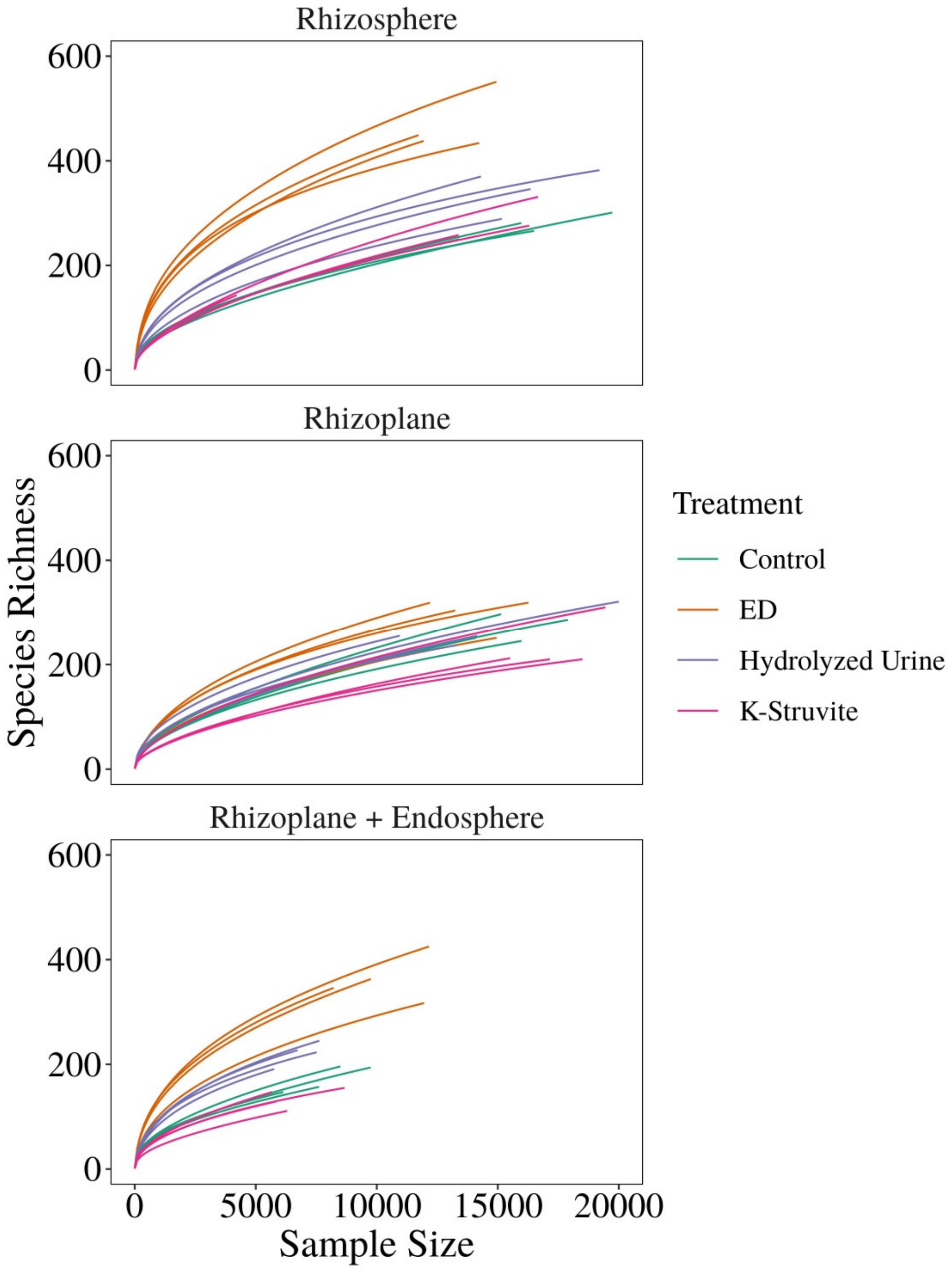

Figure S1. Rarefaction curves displaying the number of operational taxonomic units (OTU) against the depth of each of the urine-derived fertilizer samples divided by root zone (ED: electrodialysis concentrate).

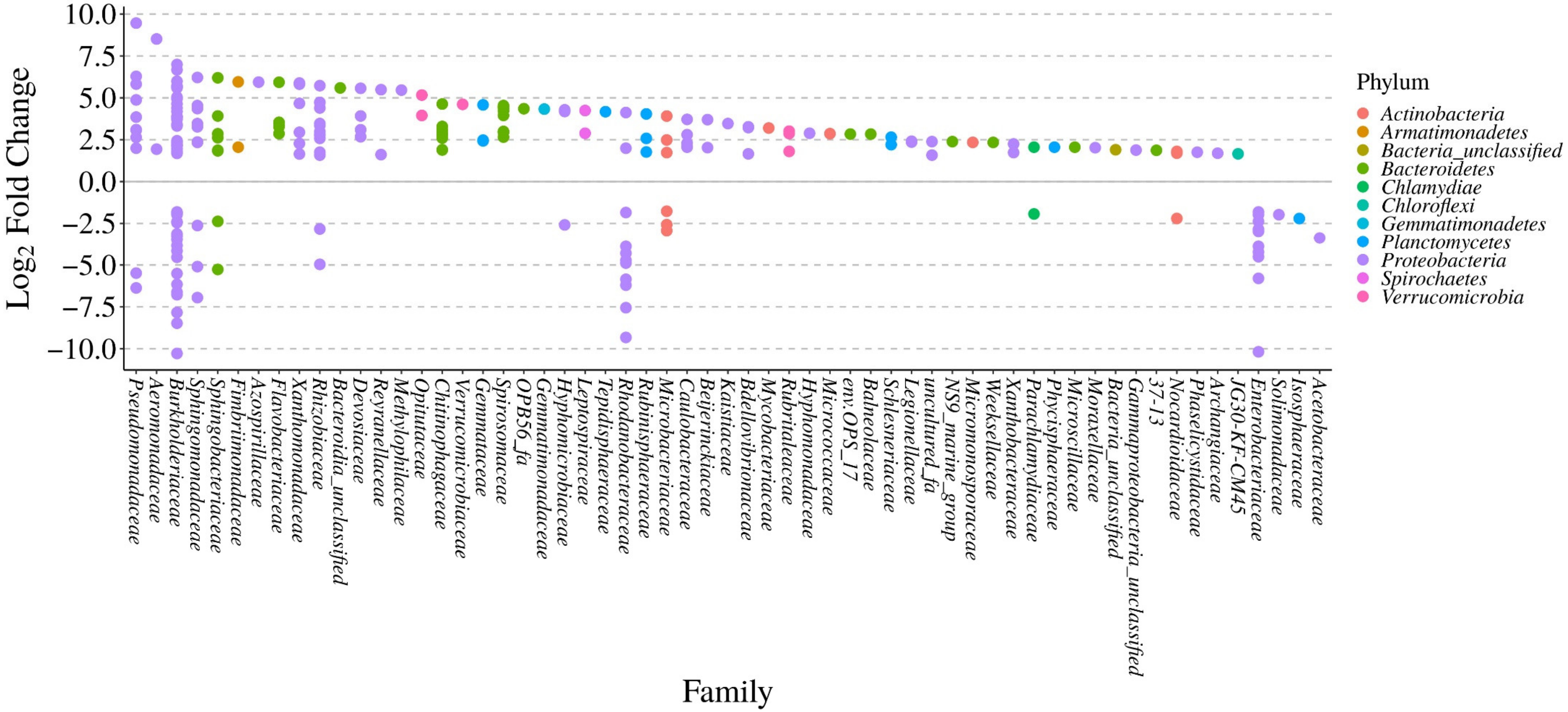

Figure S2. Differentially abundant operational taxonomic units (OTU) between the electrodialysis (ED) concentrate and NPK control-treated lettuce root-associated bacterial community samples. Taxa with positive $\log_2$ fold change values are more abundant in the ED concentrate treatment. Taxa with negative $\log_2$ fold change values are more abundant in the NPK control treatment. Significance at $\alpha = 0.01$. N per urine-derived fertilizer treatment = 12.

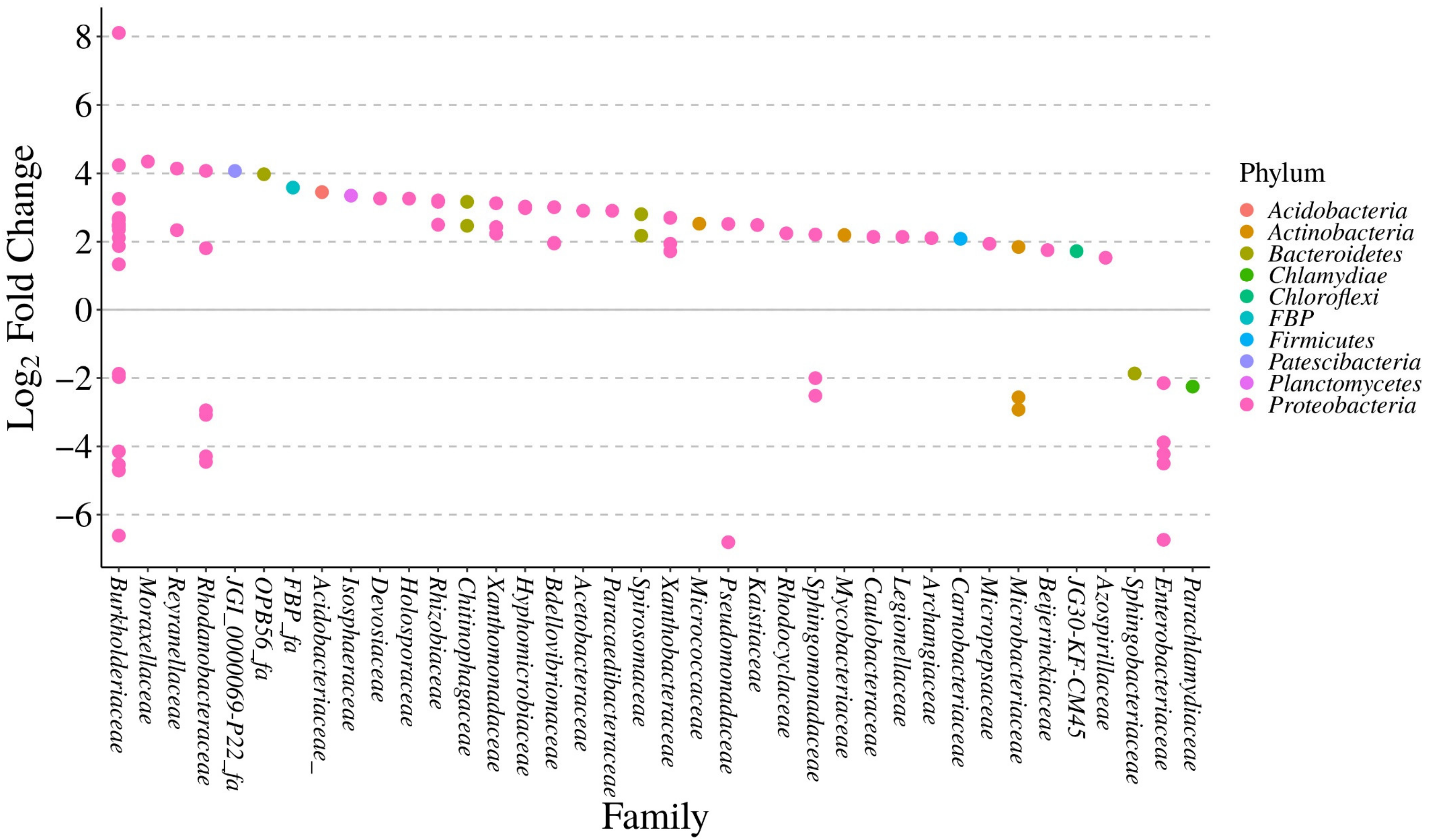

Figure S3. Differentially abundant operational taxonomic units (OTU) between the hydrolyzed urine and NPK control-treated lettuce root-associated bacterial community samples. Taxa with positive $\log_2$ fold change values are significantly more abundant in the hydrolyzed urine treatment. Taxa with negative $\log_2$ fold change values are significantly more abundant in the NPK control treatment. Significance at $\alpha = 0.01$. N per urine-derived fertilizer treatment = 12.

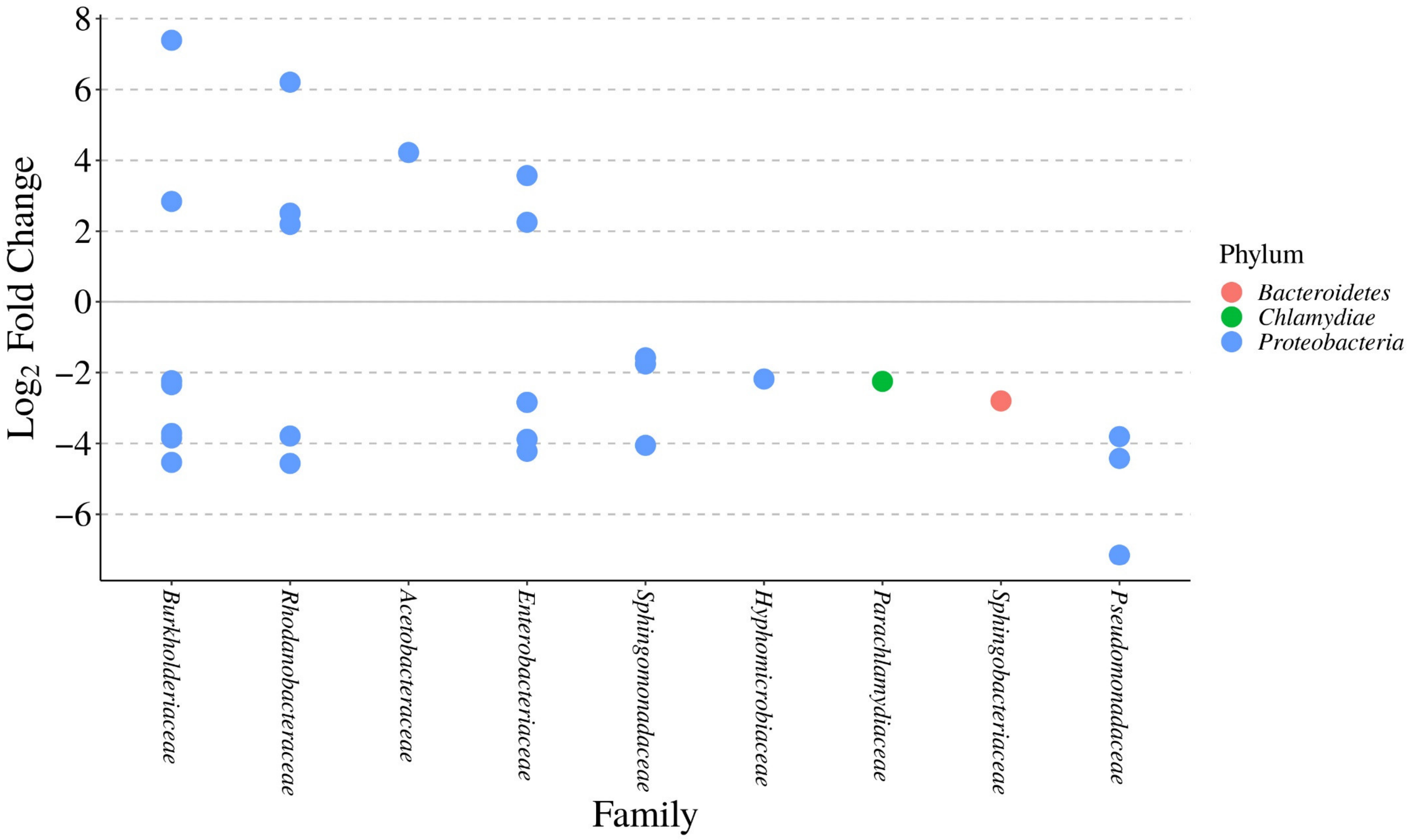

Figure S4. Differentially abundant operational taxonomic units (OTU) between the K-struvite and NPK control-treated lettuce root-associated bacterial community samples. Taxa with positive $\log_2$ fold change values are significantly more abundant in the K-struvite treatment. Taxa with negative $\log_2$ fold change values are significantly more abundant in the NPK control treatment. Significance at $\alpha = 0.01$. N per urine-derived fertilizer treatment = 12.

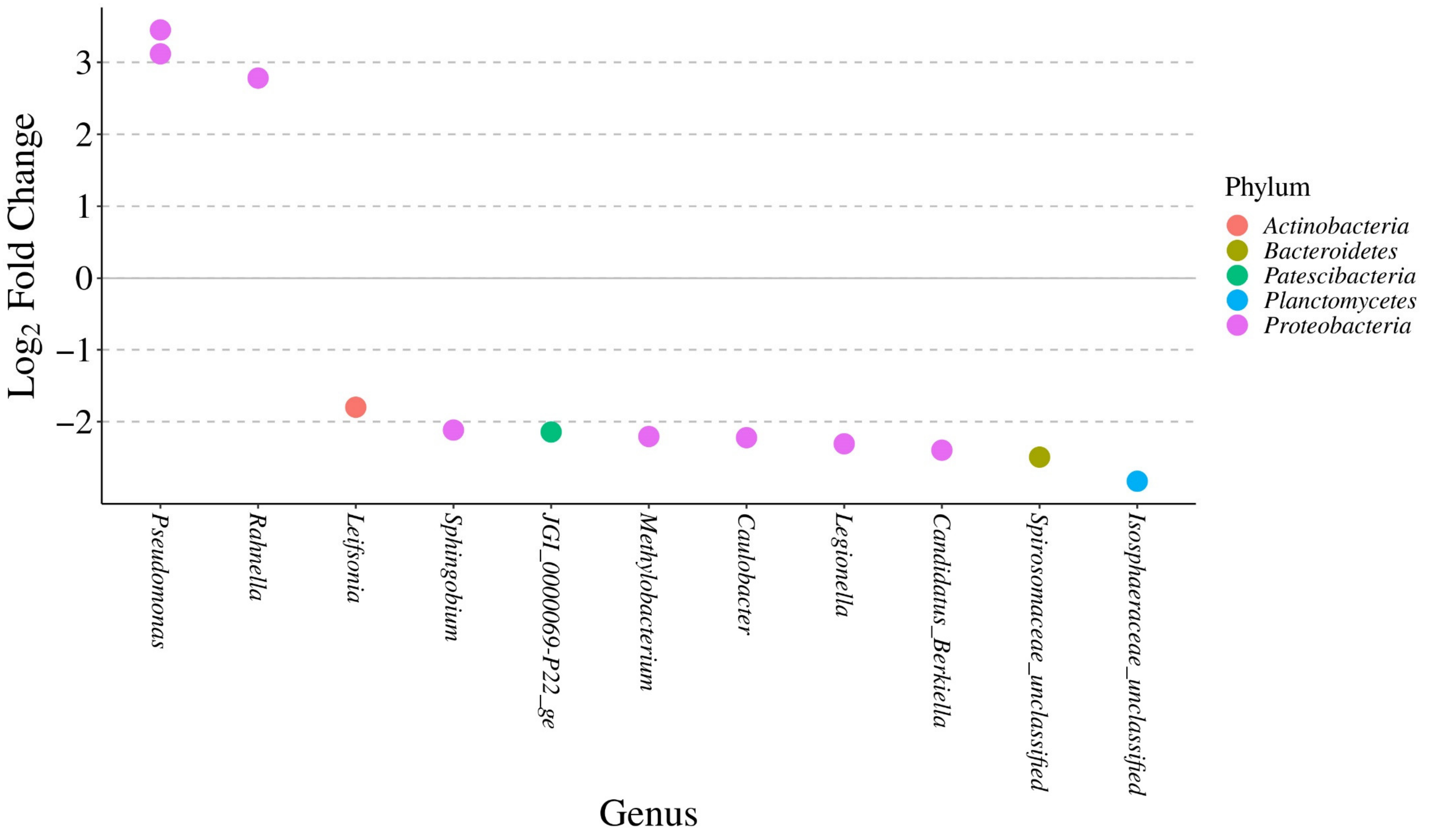

Figure S5. Differentially abundant operational taxonomic units (OTU) between the rhizoplane and rhizosphere lettuce root-associated bacterial community samples. Taxa with positive log_2 fold change values are significantly more abundant in the rhizoplane root zone. Taxa with negative log_2 fold change values are significantly more abundant in the rhizosphere root zone. Significance at $\alpha = 0.01$. n per root zone = 16.

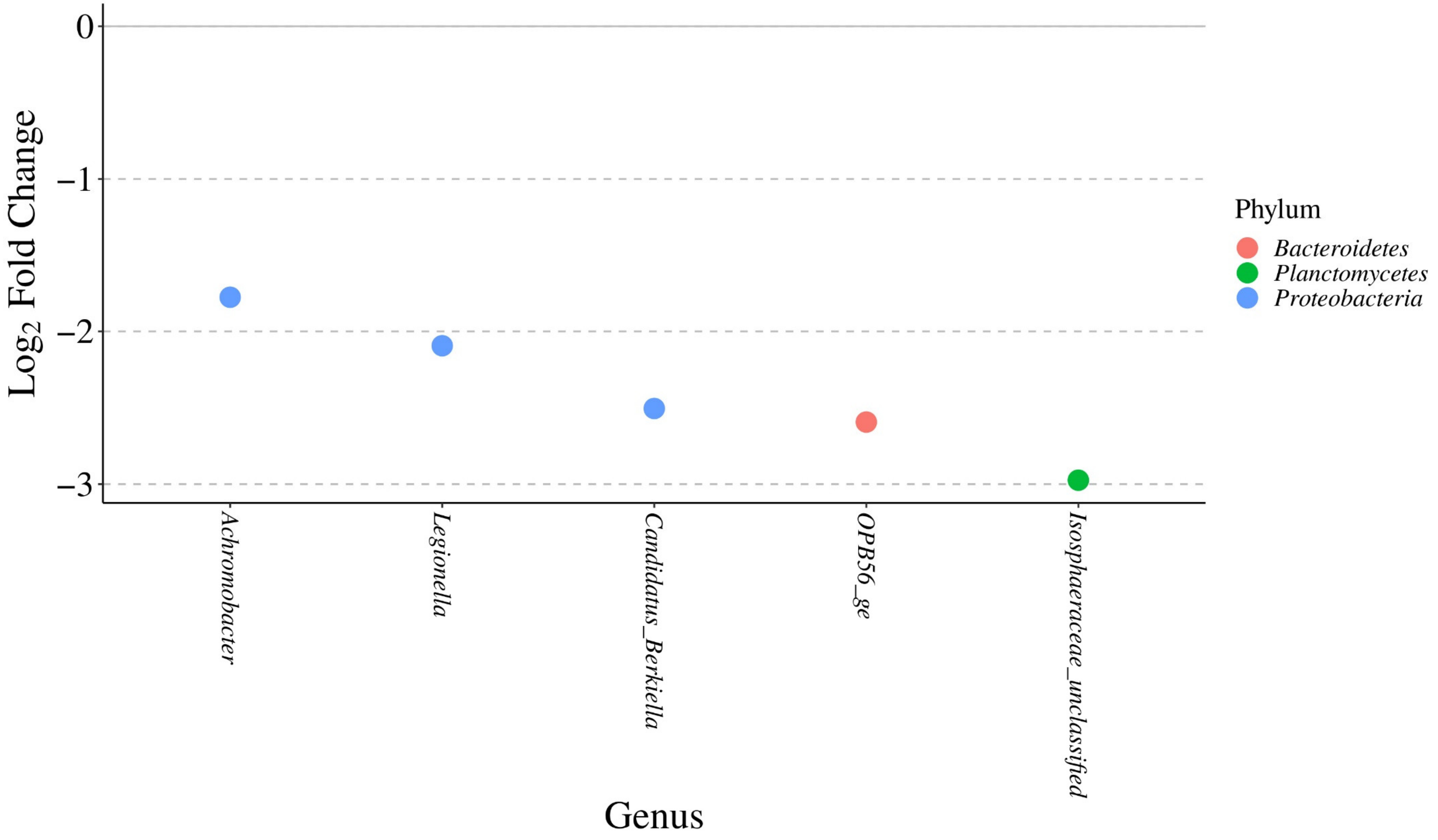

Figure S6. Differentially abundant operational taxonomic units (OTU) between the combined rhizoplane/endosphere and rhizosphere lettuce root-associated bacterial community samples. Taxa with positive $\log_2$ fold change values are significantly more abundant in the combined rhizoplane/endosphere root zone. Taxa with negative $\log_2$ fold change values are significantly more abundant in the rhizosphere root zone. Significance at $\alpha = 0.01$. n per root zone = 16.

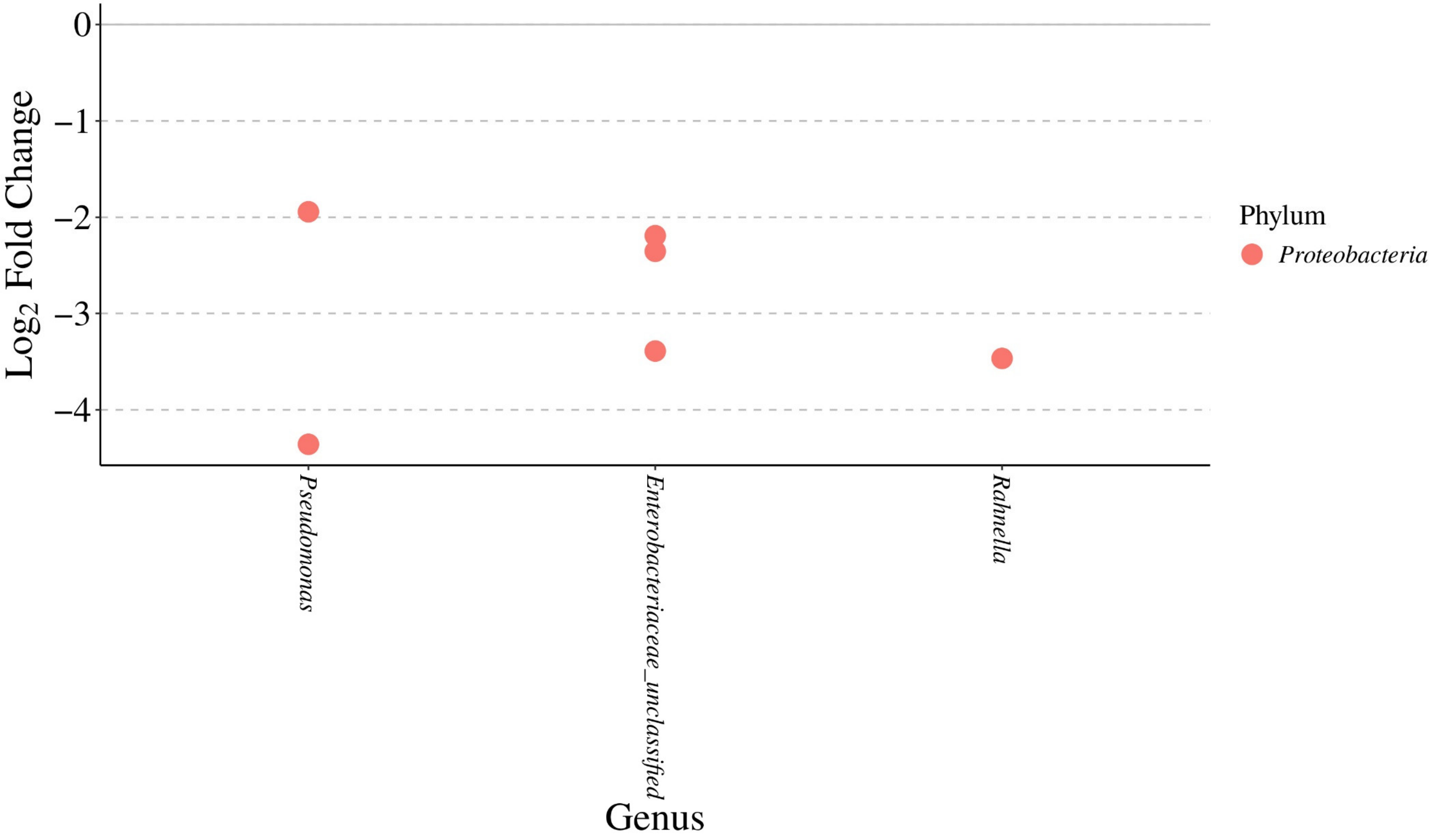

Figure S7. Differentially abundant operational taxonomic units (OTU) between the combined rhizoplane/endosphere and rhizoplane lettuce root-associated bacterial community samples. Taxa with positive $\log_2$ fold change values are significantly more abundant in the combined rhizoplane/endosphere root zone. Taxa with negative $\log_2$ fold change values are significantly more abundant in the rhizoplane root zone. Significance at $\alpha = 0.01$. n per root zone = 16.

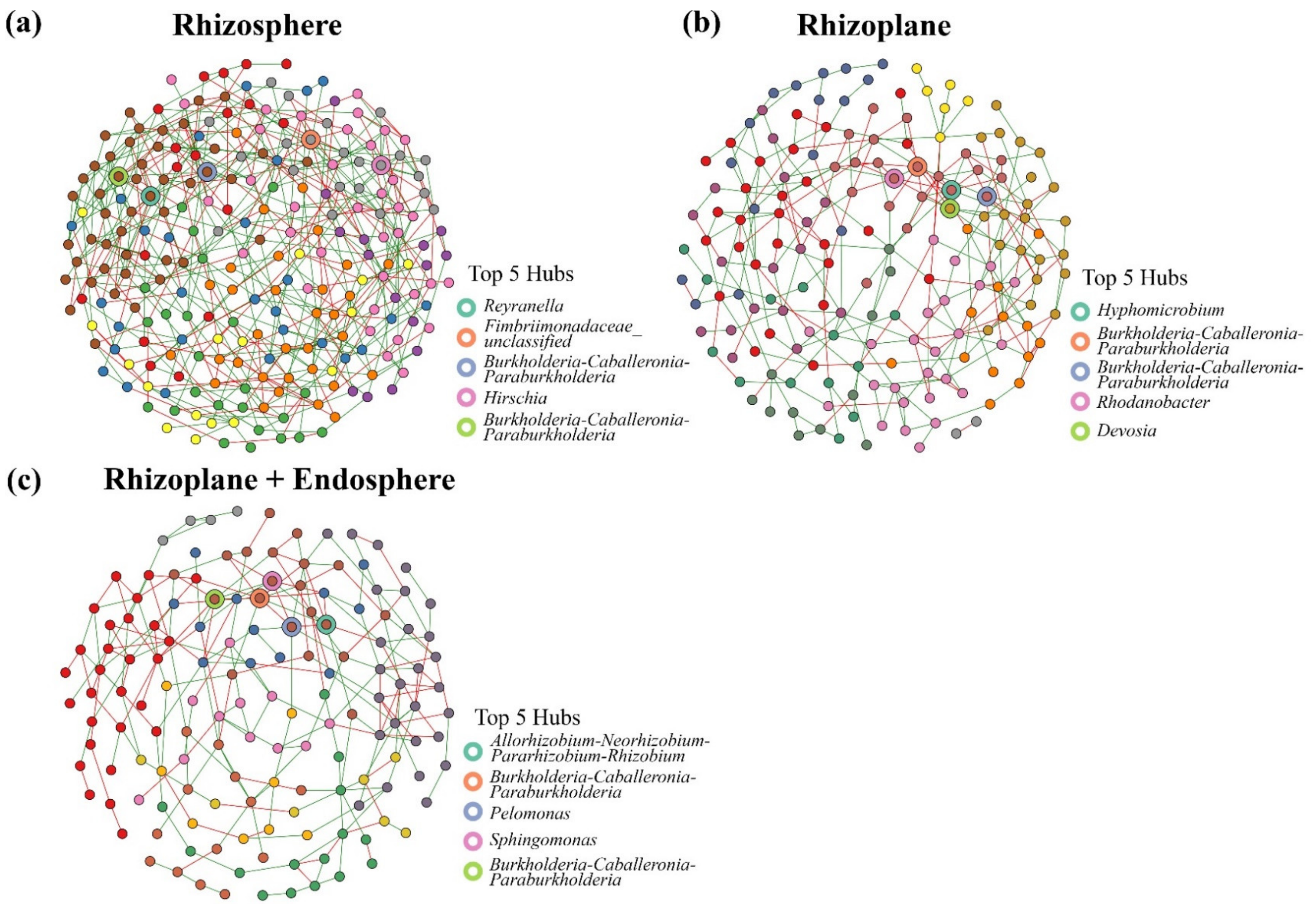

Figure S8. Lettuce root-associated bacterial community networks grouped per root zone: (a) Rhizosphere network with 237 nodes, 555 edges (367 positives and 188 negatives), and 9 clusters; (b) Rhizoplane network with 173 nodes, 301 edges (204 positives and 97 negatives), and 11 clusters; (c) Combined rhizoplane/endosphere network with 145 nodes, 237 edges (142 positives and 95 negatives), and 10 clusters. Nodes are colored by cluster (clusters were determined using the fast greedy modularity optimization algorithm). The top 5 hub nodes are indicated by the colored borders (hubs were determined using Kleinberg's hub centrality scores). The green and red edges indicate a positive or negative correlation between the network nodes, respectively.

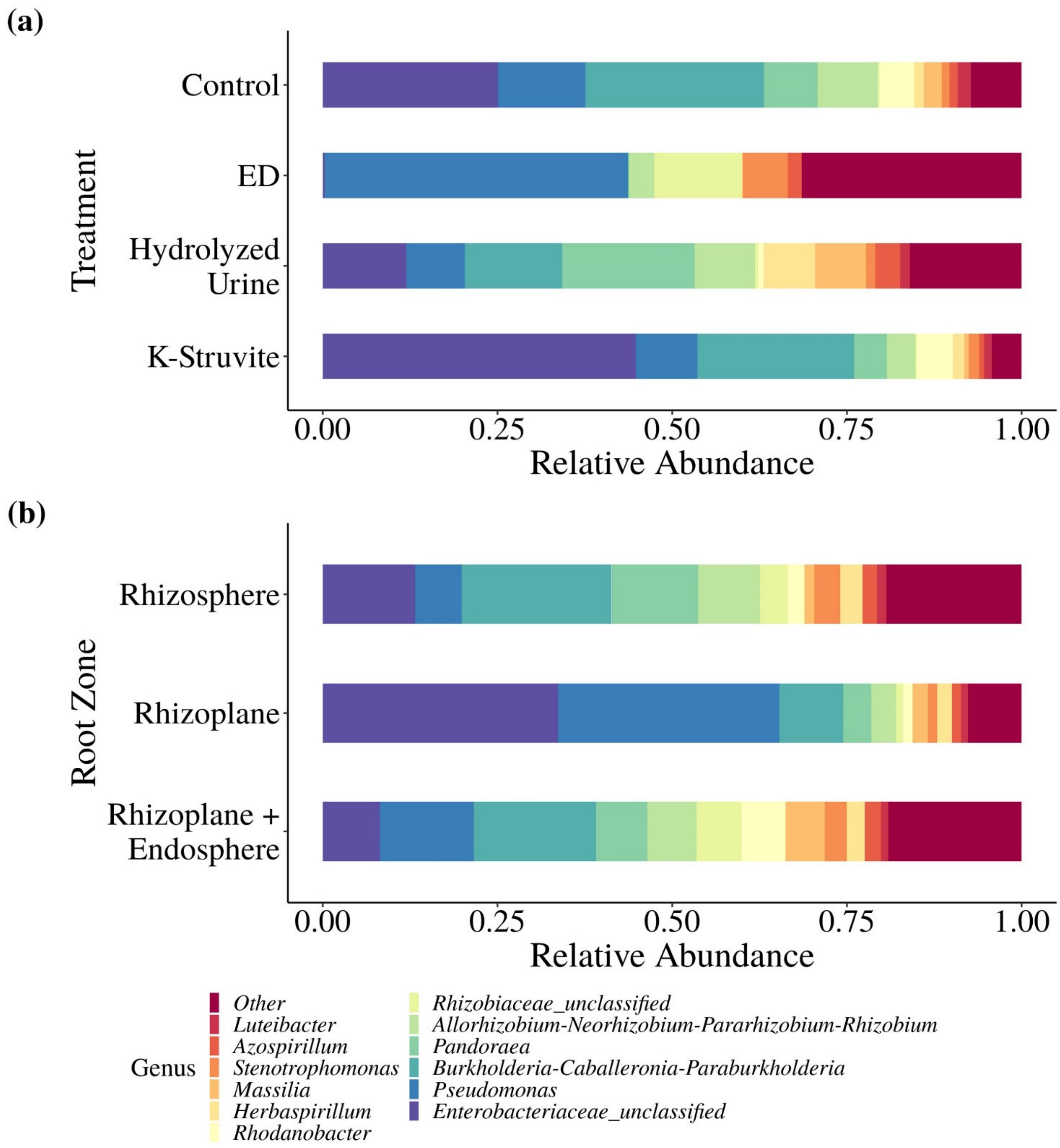

Figure S9. Relative abundance of the top 12 genera present in the lettuce root-associated bacterial community samples grouped per (a) urine-derived fertilizer treatment and (b) root zone. The top 12 genera were determined based on their combined relative abundance across all 48 root samples. All other genera were labeled as 'Other.' N per urine-derived fertilizer treatment = 12 and n per root zone = 16 (ED: electrodialysis concentrate).

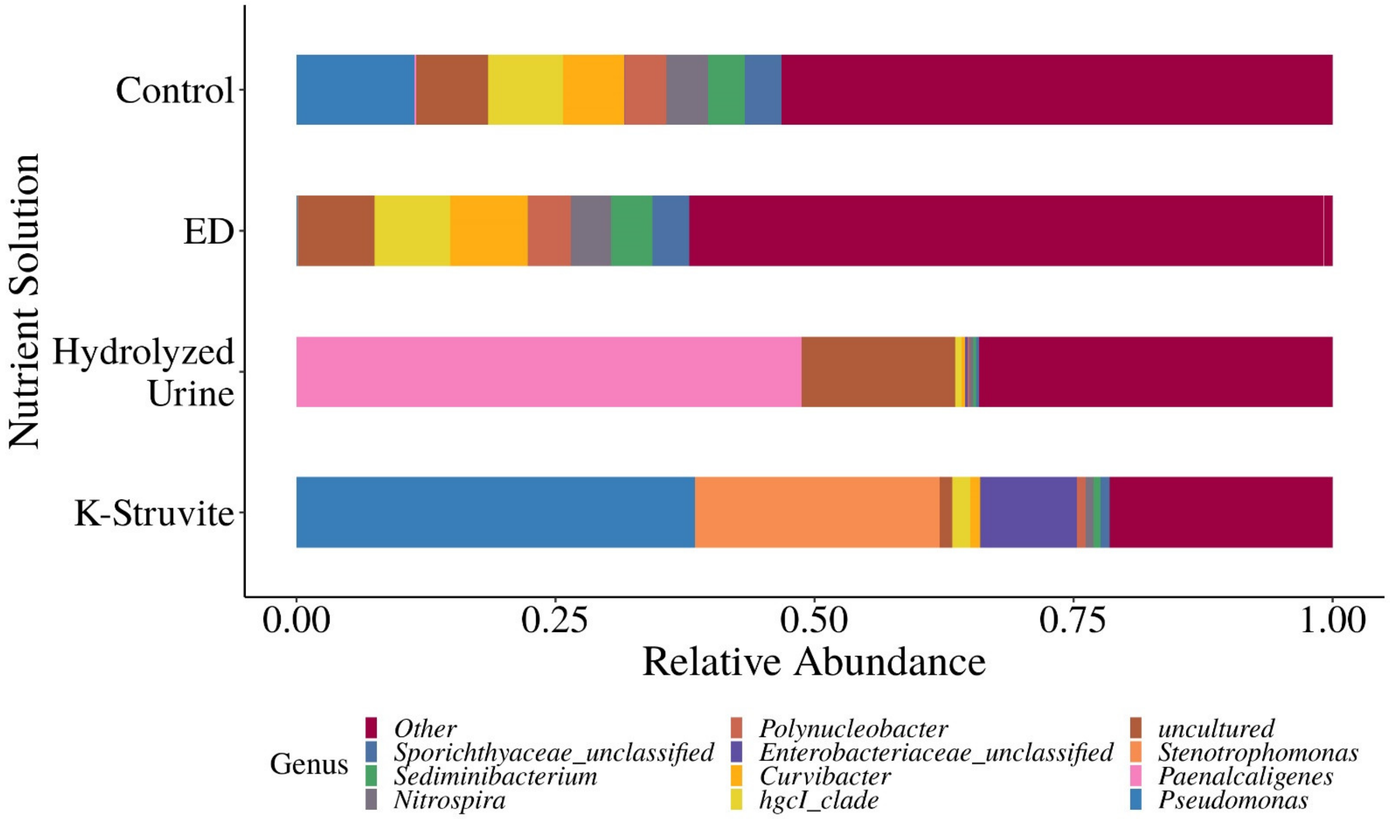

Figure S10. Relative abundance of the top 12 genera in the different urine-derived fertilizer's nutrient solutions before application. The top 12 genera were determined based on their combined relative abundance across all four nutrient solution samples. All other genera were labeled as 'Other.' n per nutrient solution = 1 (ED: electrodialysis concentrate).

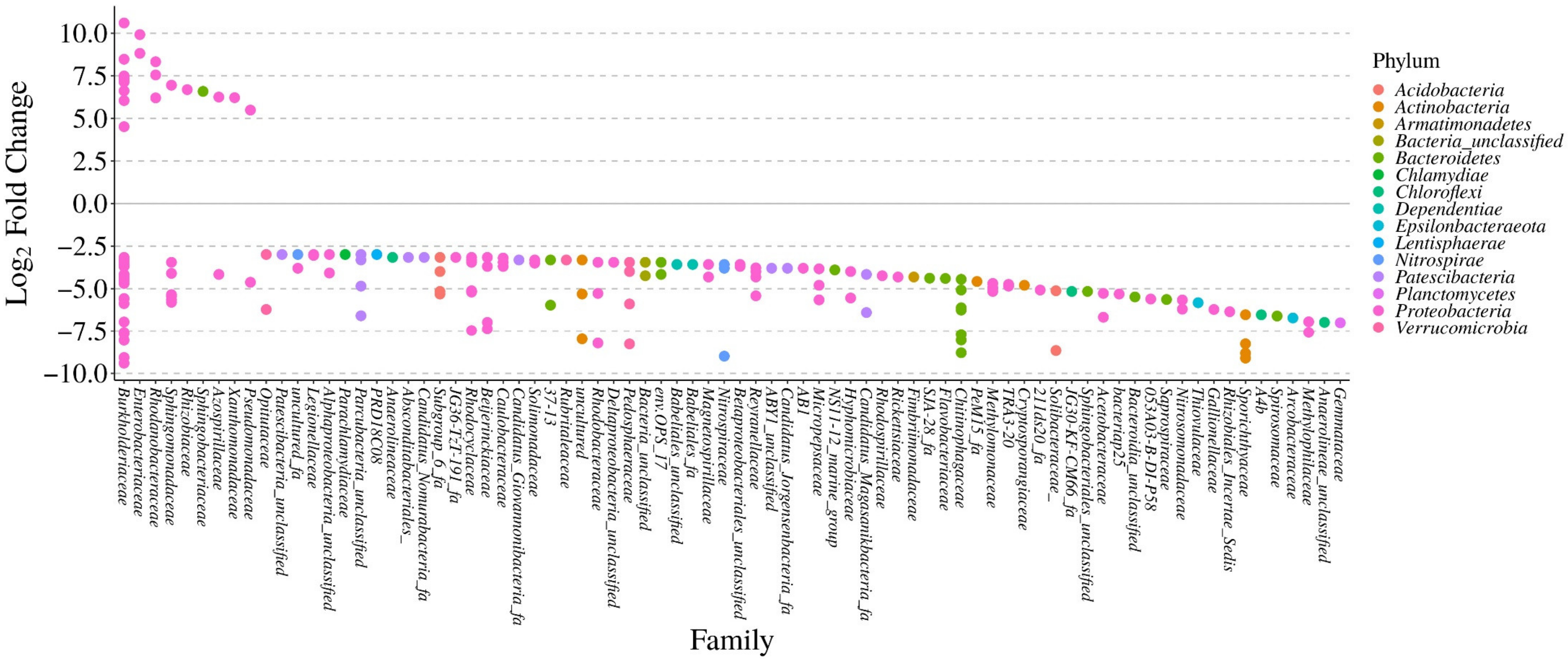

Figure S11. Differentially abundant operational taxonomic units (OTU) between the NPK control-treated lettuce root-associated bacterial community samples and the NPK control nutrient solution. Taxa with positive log_2 fold change values are more abundant in the root samples. Taxa with negative log_2 fold change values are more abundant in the nutrient solution. Significance at $\alpha = 0.01$.

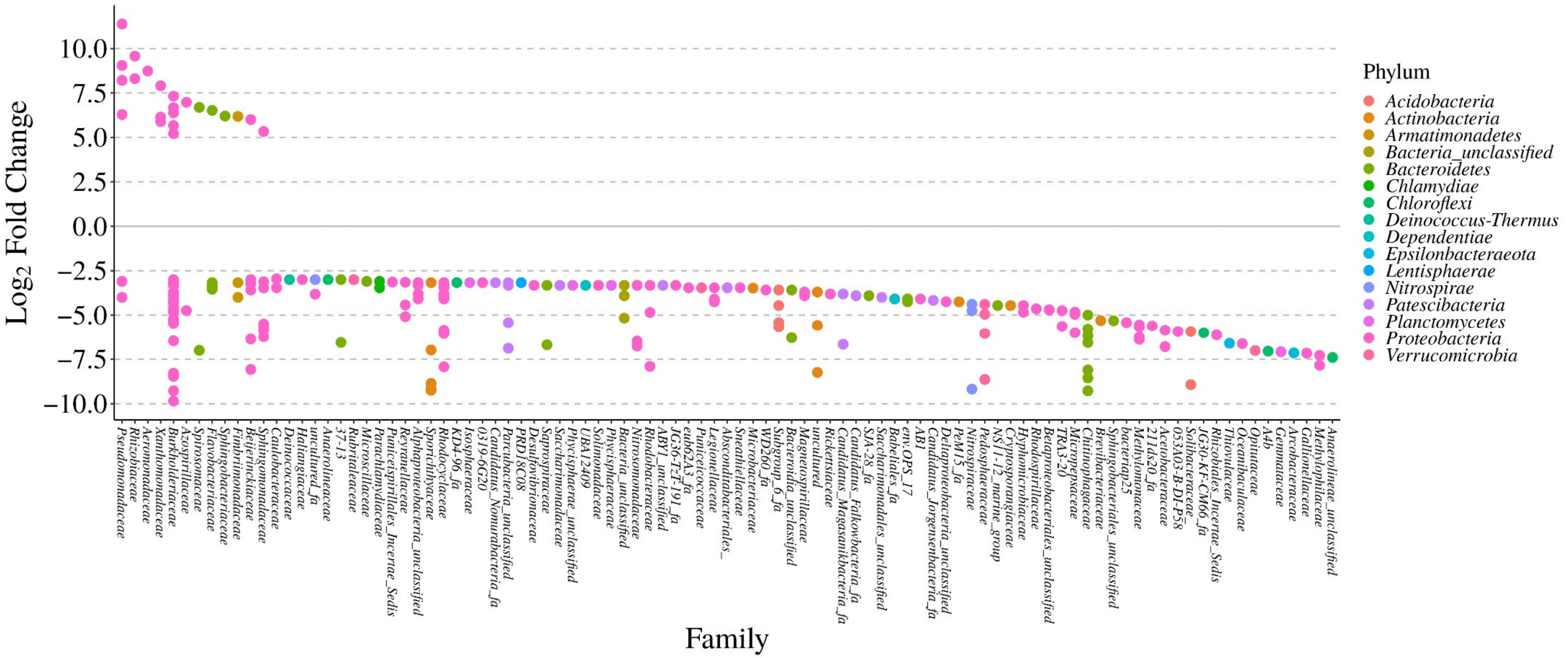

Figure S12. Differentially abundant operational taxonomic units (OTU) between the electrodialysis (ED) concentrate-treated lettuce root-associated bacterial community samples and the ED concentrate nutrient solution. Taxa with positive $\log_2$ fold change values are more abundant in the root samples. Taxa with negative $\log_2$ fold change values are more abundant in the nutrient solution. Significance at $\alpha = 0.01$.

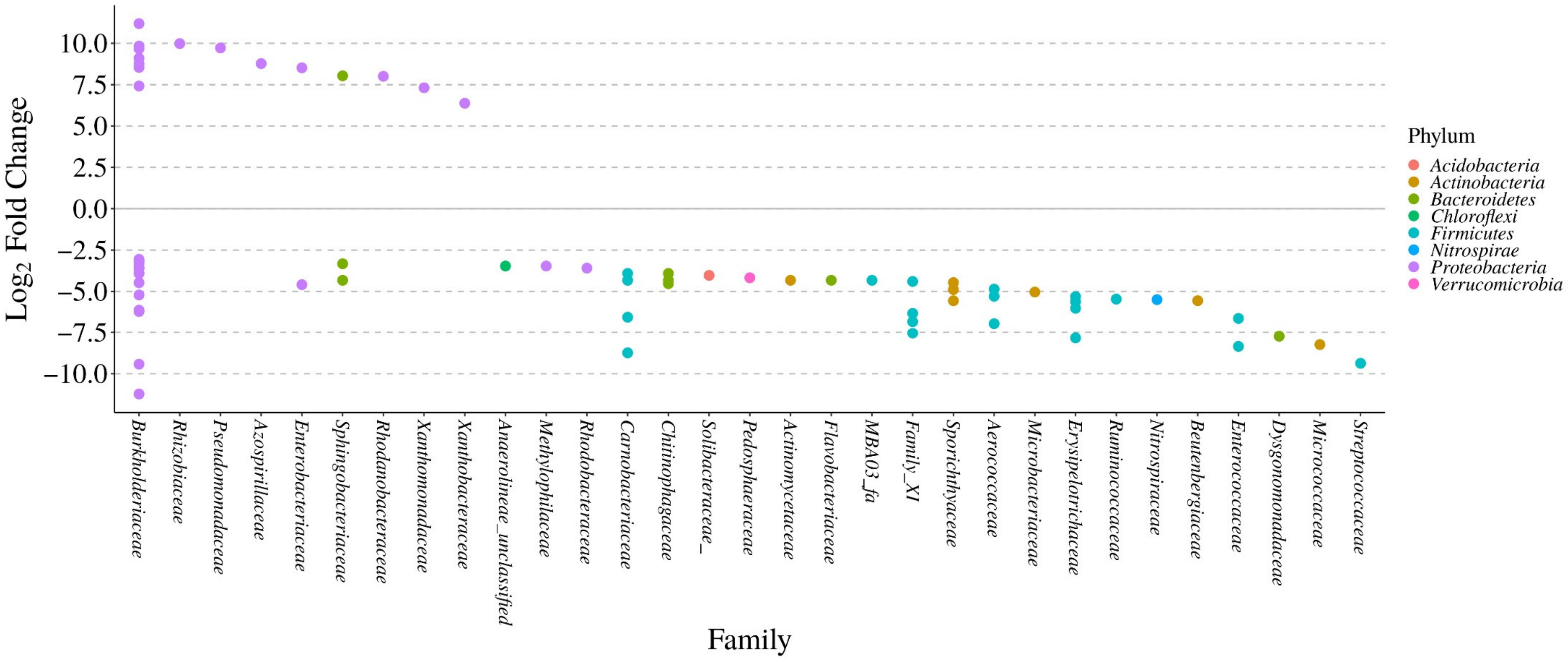

Figure S13. Differentially abundant operational taxonomic units (OTU) between the hydrolyzed urine-treated lettuce root-associated bacterial community samples and the hydrolyzed urine nutrient solution. Taxa with positive $\log_2$ fold change values are more abundant in the root samples. Taxa with negative $\log_2$ fold change values are more abundant in the nutrient solution. Significance at $\alpha = 0.01$.

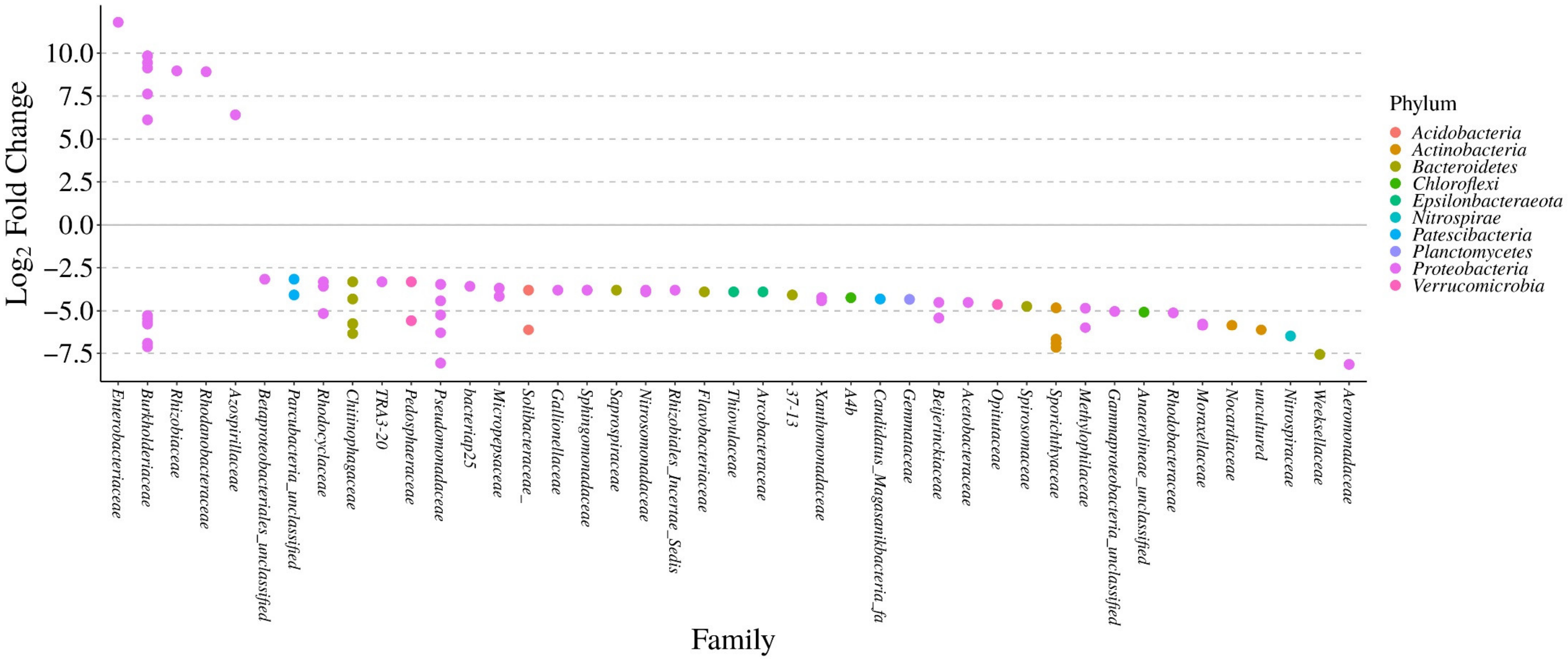

Figure S14. Differentially abundant operational taxonomic units (OTU) between the K-struvite-treated lettuce root-associated bacterial community samples and the K-struvite nutrient solution. Taxa with positive log_2 fold change values are more abundant in the root samples. Taxa with negative log_2 fold change values are more abundant in the nutrient solution. Significance at $\alpha = 0.01$.

Tables

Table S1. Lettuce leaf number, leaf area, fresh weight (FW), dry weight (DW), and dry matter content (% DM) regarding the urine-derived fertilizer treatments. Asterisks indicate global level of significance: $p < 0.001$ (***). Different letters within each column indicate significant differences according to Duncan's multiple range test ($\alpha = 0.05$). All data are expressed as mean ± standard error, n = 4 (ED: electrodialysis, TE: trace elements).

Treatments	Leaf Number (no. plant^{-1})	Leaf Area (cm^2.plant^{-1})	FW (g.plant^{-1})	DW (g.plant^{-1})	Dry Matter Content (% DM)
NPK control (20-10-20 + TE)	15 ± 0.37 ab	1178 ± 23.31 a	43.25 ± 1.36 a	3.22 ± 0.07 a	7.47 ± 0.16 b
ED Concentrate	14 ± 0.34 b	841 ± 13.98 b	30.16 ± 0.72 b	2.33 ± 0.04 b	7.72 ± 0.17 b
Hydrolyzed Urine	9 ± 0.53 c	283 ± 17.82 c	8.30 ± 0.95 c	0.73 ± 0.10 c	8.72 ± 0.20 a
K-Struvite	16 ± 0.64 a	1114 ± 33.71 a	41.96 ± 0.34 a	3.08 ± 0.08 a	7.35 ± 0.13 b
Significance	***	***	***	***	***

Table S2. Lettuce leaf photosynthetic pigments and stress metabolites in respect to the urine-derived fertilizer treatments. The photosynthetic pigment parameters are SPAD index, Chlorophyll a (Chl_a), chlorophyll b (Chl_b), total chlorophylls (Chl_{a+b}), and total carotenoids. The stress metabolite parameters are total antioxidant capacity (TAC) and total phenolic content (TPC). Asterisks indicate global level of significance: $p < 0.05$ (*) and $p < 0.001$ (***). Different letters within each column indicate significant differences according to Duncan's multiple range test ($\alpha = 0.05$). All data are expressed as mean ± standard error, n = 3 to 4. FW = fresh weight, L-AAE = L-ascorbic acid equivalents, and GAE = gallic acid equivalents (ED: electrodialysis, TE: trace elements).

Treatments	SPAD Index	Chl_a ($mg.kg^{-1}$ FW)	Chl_b ($mg.kg^{-1}$ FW)	Chl_{a+b} ($mg.kg^{-1}$ FW)	Total Carotenoids ($mg.kg^{-1}$ FW)	TAC (µmol L-AAE.$100g^{-1}$ FW)	TPC (µg GAE.$100g^{-1}$ FW)
NPK control (20-10-20 + TE)	24.95 ± 0.30 b	148.1 ± 2.1 ab	45.90 ± 1.2 a	194.0 ± 3.3 ab	44.30 ± 0.7 ab	31.46 ± 0.23 c	836.5 ± 10 b
ED Concentrate	20.33 ± 0.34 c	115.4 ± 4.4 b	31.27 ± 1.0 b	146.6 ± 4.6 b	36.03 ± 1.9 b	35.32 ± 0.28 a	1083.4 ± 18 a
Hydrolyzed Urine	26.88 ± 0.33 a	177.1 ± 20.4 a	47.93 ± 5.3 a	225.1 ± 25.7 a	54.07 ± 5.8 a	33.27 ± 0.15 b	863.4 ± 25 b
K-Struvite	23.90 ± 0.41 b	145.8 ± 7.3 ab	42.67 ± 2.1 a	188.5 ± 9.4 ab	45.77 ± 2.5 ab	28.71 ± 0.79 d	881 ± 16 b
Significance	***	*	*	*	*	***	***

Table S3. Lettuce organic acid content in respect to the urine-derived fertilizer treatments. Asterisks indicate global level of significance: $p < 0.01$ (**) and $p < 0.001$ (***). Different letters within each column indicate significant differences according to Duncan's multiple range test ($\alpha = 0.05$). All data are expressed as mean ± standard error, n = 4. DW = dry weight (ED: electrodialysis, TE: trace elements).

Treatments	Malate ($mg.kg^{-1}$ DW)	Tartrate ($mg.kg^{-1}$ DW)	Oxalate ($mg.kg^{-1}$ DW)	Citrate ($mg.kg^{-1}$ DW)	Isocitrate ($mg.kg^{-1}$ DW)
NPK control (20-10-20 + TE)	23.09 ± 1.00 b	2.70 ± 0.19 a	1.48 ± 0.02 b	7.32 ± 0.36 b	0.15 ± 0.01 bc
ED Concentrate	65.55 ± 5.09 a	3.21 ± 0.30 a	2.07 ± 0.25 a	17.91 ± 1.43 a	0.51 ± 0.04 a
Hydrolyzed Urine	3.25 ± 0.15 c	1.69 ± 0.21 b	0.71 ± 0.05 c	2.85 ± 0.59 c	0.08 ± 0.02 c
K-struvite	18.31 ± 2.21 b	2.54 ± 0.25 a	1.68 ± 0.09 ab	8.07 ± 0.84 b	0.16 ± 0.02 b
Significance	***	**	***	***	***

Table S4. Lettuce leaf mineral analysis in respect to the urine-derived fertilizer treatments. Asterisks indicate global level of significance: $p < 0.001$ (***). Different letters within each column indicate significant differences according to Duncan's multiple range test (α = 0.05). All data are expressed as mean ± standard error, n = 4. FW = fresh weight and DW = dry weight (ED: electrodialysis, TE: trace elements).

**Treatments**	**Total N (% DW)**	**NO_3^- ($mg.kg^{-1}$ FW)**	**NH_4^+ ($mg.kg^{-1}$ DW)**	**PO_4^{3-} ($g.kg^{-1}$ DW)**	**K^+ ($g.kg^{-1}$ DW)**	**Ca^{2+} ($g.kg^{-1}$ DW)**	**Mg^{2+} ($g.kg^{-1}$ DW)**	**Na^+ ($g.kg^{-1}$ DW)**	**Cl^- ($g.kg^{-1}$ DW)**	**SO_4^{2-} ($g.kg^{-1}$ DW)**
NPK control (20-10-20 + TE)	4.93 ± 0.03 b	3,162 ± 158 b	0.53 ± 0.04 b	22.63 ± 0.57 a	62.14 ± 0.73 a	3.10 ± 0.33 a	3.87 ± 0.24 b	2.19 ± 0.35 d	2.40 ± 0.16 c	3.26 ± 0.38 c
ED Concentrate	3.60 ± 0.10 c	2,160 ± 86 c	0.45 ± 0.03 b	8.09 ± 0.37 d	56.13 ± 2.52 b	1.70 ± 0.16 b	1.43 ± 0.11 c	31.05 ± 0.80 a	17.55 ± 0.29 b	2.94 ± 0.18 c
Hydrolyzed Urine	5.35 ± 0.12 a	44 ± 8 d	1.26 ± 0.19 a	10.01 ± 0.75 c	31.34 ± 2.49 c	1.20 ± 0.16 b	1.92 ± 0.38 c	4.30 ± 0.43 c	37.63 ± 2.25 a	11.72 ± 1.30 a
K-Struvite	4.99 ± 0.10 b	4,463 ± 117 a	0.95 ± 0.05 a	20.68 ± 0.22 b	50.64 ± 0.73 b	1.64 ± 0.07 b	6.42 ± 0.35 a	14.09 ± 0.85 b	3.26 ± 0.27 c	9.35 ± 0.58 b
Significance	***	***	***	***	***	***	***	***	***	***

Table S5. PerMANOVA with Holm adjustment for multiple comparisons on Bray-Curtis distances to determine differences in lettuce root-associated bacterial community composition (β-diversity) and homogeneity of variances explained by the urine-derived fertilizers. Asterisks indicate level of significance: $p < 0.05$ (*), $p < 0.01$ (**), and $p < 0.0001$ (****). n.s. = non significant (ED: electrodialysis concentrate).

		β-diversity			Homogeneity of Variances
		F	R^2	*p*	
Global		18.78	0.56	****	n.s.
Pairwise	NPK control - ED	33.64	0.60	**	n.s.
	NPK control - Hydrolyzed Urine	11.00	0.33	**	n.s.
	NPK control - K-Struvite	3.27	0.13	*	*
	ED - Hydrolyzed Urine	25.67	0.54	**	n.s.
	ED - K-Struvite	27.27	0.55	**	n.s.
	Hydrolyzed Urine - K-Struvite	11.90	0.35	**	n.s.

Table S6. PerMANOVA with Holm adjustment for multiple comparisons on Bray-Curtis distances to determine differences in lettuce root-associated bacterial community composition (β-diversity) and homogeneity of variances explained by root zone. Asterisks indicate level of significance: $p < 0.01$ (**) and $p < 0.001$ (***). n.s. = non significant.

		β-diversity			Homogeneity of Variances
		F	R^2	*p*	
Global		3.94	0.15	***	n.s.
Pairwise	Rhizosphere - Rhizoplane	4.58	0.13	**	n.s.
	Rhizosphere - Rhizoplane + Endosphere	2.16	0.07	n.s.	n.s.
	Rhizoplane - Rhizoplane + Endosphere	5.15	0.15	**	n.s.

Table S7. Indicator operational taxonomic units (OTU) for the lettuce root-associated bacterial community NPK control samples ordered by the indicator value. Multi-level pattern analysis (permutations = 10,000) with the "IndVal.g" indicator value index was utilized to identify the indicator OTUs. The specificity (A) indicates the probability that the sample belongs to the NPK control group, given that the OTU has been found in the sample. The fidelity (B) indicates the probability of finding the OTU in samples belonging to the NPK control group. P-values were adjusted for multiple testing with the Benjamini & Hochberg adjustment. Asterisks indicate level of significance: $p < 0.05$ (*) and $p < 0.01$ (**). Total n = 48 and n per treatment group = 12.

Family	Genus	A (%)	B (%)	Indicator Value	Significance
Pseudomonadaceae	*Pseudomonas*	97.74	100	0.99	**
Rhodanobacteraceae	*Rhodanobacteraceae_unclassified*	86.29	91.67	0.89	**
Enterobacteriaceae	*Pantoea*	90.91	83.33	0.87	**
Burkholderiaceae	*Pandoraea*	100	75	0.87	**
Sphingobacteriaceae	*Mucilaginibacter*	78.13	91.67	0.85	**
Enterobacteriaceae	*Enterobacteriaceae_unclassified*	100	66.67	0.82	**
Parachlamydiaceae	*Candidatus_Protochlamydia*	93.75	58.33	0.74	**
Sphingomonadaceae	*Sphingomonadaceae_unclassified*	91.67	58.33	0.73	**
Burkholderiaceae	*Burkholderia-Caballeronia-Paraburkholderia*	100	50	0.71	**
Sphingobacteriaceae	*Mucilaginibacter*	100	50	0.71	**
Methylophilaceae	*Methylophilus*	78.13	58.33	0.68	*
Pseudomonadaceae	*Pseudomonas*	100	41.67	0.65	*
Burkholderiaceae	*Burkholderia-Caballeronia-Paraburkholderia*	82.35	50	0.64	*
*Acidobacteriaceae_*	*Granulicella*	98.25	41.67	0.64	*

Table S8. Top 20 indicator operational taxonomic units (OTU) for the lettuce root-associated bacterial community electrodialysis (ED) concentrate samples ordered by the indicator value. Multi-level pattern analysis (permutations = 10,000) with the "IndVal.g" indicator value index was utilized to identify the indicator OTUs. The specificity (A) indicates the probability that the sample belongs to the ED group, given that the OTU has been found in the sample. The fidelity (B) indicates the probability of finding the OTU in samples belonging to the ED group. P-values were adjusted for multiple testing with the Benjamini & Hochberg adjustment. Asterisks indicate level of significance: $p < 0.01$ (**). Total n = 48 and n per treatment group = 12.

Family	Genus	A (%)	B (%)	Indicator Value	Significance
Devosiaceae	*Devosia*	100	100	1	**
Gemmatimonadaceae	*Gemmatimonas*	100	100	1	**
Leptospiraceae	*Leptospira*	100	100	1	**
Rhizobiaceae	*Aminobacter*	100	100	1	**
Rubinisphaeraceae	*SH-PL14*	100	100	1	**
Sphingomonadaceae	*Sphingomonas*	100	100	1	**
Spirosomaceae	*Arcicella*	100	100	1	**
Spirosomaceae	*Flectobacillus*	100	100	1	**
Xanthomonadaceae	*Pseudoxanthomonas*	100	100	1	**
Pseudomonadaceae	*Pseudomonas*	99.90	100	0.999	**
Sphingobacteriaceae	*Pedobacter*	99.89	100	0.999	**
Xanthomonadaceae	*Pseudoxanthomonas*	99.86	100	0.999	**
Methylophilaceae	*Methylophilus*	99.81	100	0.999	**
Aeromonadaceae	*Aeromonas*	99.63	100	0.998	**
Burkholderiaceae	*Pelomonas*	99.51	100	0.998	**
Burkholderiaceae	*Acidovorax*	99.48	100	0.997	**
Burkholderiaceae	*Burkholderiaceae_unclassified*	99.44	100	0.997	**
Burkholderiaceae	*Acidovorax*	99.44	100	0.997	**
Pseudomonadaceae	*Pseudomonas*	99.35	100	0.997	**
Spirosomaceae	*Emticicia*	99.20	100	0.996	**

Table S9. Top 20 indicator operational taxonomic units (OTU) for the lettuce root-associated bacterial community hydrolyzed urine samples ordered by the indicator value. Multi-level pattern analysis (permutations = 10,000) with the "IndVal.g" indicator value index was utilized to identify the indicator OTUs. The specificity (A) indicates the probability that the sample belongs to the hydrolyzed urine group, given that the OTU has been found in the sample. The fidelity (B) indicates the probability of finding the OTU in samples belonging to the hydrolyzed urine group. P-values were adjusted for multiple testing with the Benjamini & Hochberg adjustment. Asterisks indicate level of significance: $p < 0.01$ (**). Total n = 48 and n per treatment group = 12.

Family	Genus	A (%)	B (%)	Indicator Value	Significance
Burkholderiaceae	*Burkholderiaceae_unclassified*	98.32	100	0.99	**
*Acidobacteriaceae_*	*Edaphobacter*	94.69	100	0.97	**
Micropepsaceae	*uncultured*	97.44	91.67	0.95	**
Burkholderiaceae	*Eoetvoesia*	92.54	91.67	0.92	**
Rhodocyclaceae	*Zoogloea*	100	83.33	0.91	**
JGI_0000069-P22_fa	*JGI_0000069-P22_ge*	88.32	91.67	0.90	**
*Solibacteraceae_*	*Paludibaculum*	96	83.33	0.89	**
Burkholderiaceae	*Alicycliphilus*	83.58	91.67	0.88	**
Carnobacteriaceae	*uncultured*	100	75.00	0.87	**
Rhodanobacteraceae	*Rhodanobacter*	72.83	100	0.85	**
Holosporaceae	*uncultured*	86.67	83.33	0.85	**
Burkholderiaceae	*Paenalcaligenes*	94.12	75	0.84	**
Moraxellaceae	*Acinetobacter*	93.55	75	0.84	**
Burkholderiaceae	*Burkholderiaceae_unclassified*	92.98	75	0.84	**
FBP_fa	*FBP_ge*	92.31	75	0.83	**
Burkholderiaceae	*Pigmentiphaga*	81.82	83.33	0.83	**
Rhodanobacteraceae	*Rudaea*	100	66.67	0.82	**
Burkholderiaceae	*Burkholderia-Caballeronia-Paraburkholderia*	96.77	66.67	0.80	**
Bdellovibrionaceae	*Bdellovibrio*	92.11	66.67	0.78	**
Chitinophagaceae	*Sediminibacterium*	100	58.33	0.76	**

Table S10. Indicator operational taxonomic units (OTU) for the lettuce root-associated bacterial community K-struvite samples ordered by the indicator value. Multi-level pattern analysis (permutations = 10,000) with the "IndVal.g" indicator value index was utilized to identify the indicator OTUs. The specificity (A) indicates the probability that the sample belongs to the K-Struvite group, given that the OTU has been found in the sample. The fidelity (B) indicates the probability of finding the OTU in samples belonging to the K-Struvite group. P-values were adjusted for multiple testing with the Benjamini & Hochberg adjustment. Asterisks indicate level of significance: $p < 0.05$ (*) and $p < 0.01$ (**). Total n = 48 and n per treatment group = 12.

Family	Genus	A (%)	B (%)	Indicator Value	Significance
Enterobacteriaceae	*Enterobacteriaceae_unclassified*	96.45	100	0.98	**
Burkholderiaceae	*Thiomonas*	92.50	100	0.96	**
Rhodanobacteraceae	*Rhodanobacter*	99.39	75	0.86	**
Enterobacteriaceae	*Enterobacteriaceae_unclassified*	98.04	58.33	0.76	**
Xanthomonadaceae	*Stenotrophomonas*	90	58.33	0.72	**
Rhodanobacteraceae	*Rhodanobacteraceae_unclassified*	94.55	50	0.69	**
Gammaproteobacteria_unclassified	*Gammaproteobacteria_unclassified*	100	41.67	0.65	*
Burkholderiaceae	*Burkholderiaceae_unclassified*	80	50	0.63	*
Enterobacteriaceae	*Enterobacteriaceae_unclassified*	90	41.67	0.61	*

Table S11. The top 10 genera ordered by node degree in the lettuce root-associated bacterial community networks grouped per urine-derived fertilizer treatment. The degree of a node is the number of adjacent edges (ED: electrodialysis).

	**NPK Control**		**ED Concentrate**		**Hydrolyzed Urine**		**K-Struvite**	
#	**Genus**	**Degree**	**Genus**	**Degree**	**Genus**	**Degree**	**Genus**	**Degree**
1	*Rhodanobacteraceae_ unclassified*	6	*Cupriavidus*	11	*Sphingobium*	8	*Allorhizobium-Neorhizobium-Pararhizobium-Rhizobium*	5
2	*Enterobacteriaceae_ unclassified*	5	*Burkholderiaceae_ unclassified*	11	*JGI_0000069-P22_ge*	8	*Herbaspirillum*	5
3	*Pandoraea*	5	*Chlamydiales_ unclassified*	11	*Enterobacteriaceae_ unclassified*	7	*Sphingomonas*	5
4	*Pseudomonas*	5	*Massilia*	10	*Stenotrophomonas*	7	*Enterobacteriaceae_ unclassified*	5
5	*Cupriavidus*	5	*Azospirillum*	10	*Massilia*	7	*Burkholderia-Caballeronia-Paraburkholderia*	4
6	*Hyphomicrobium*	5	*Fimbriimonadaceae_ unclassified*	10	uncultured *Holosporaceae*	7	*Luteibacter*	4
7	*Pseudomonas*	5	*Methylophilus*	10	*Rhodopseudomonas*	7	*Cupriavidus*	4
8	*Burkholderia-Caballeronia-Paraburkholderia*	5	*Kaistia*	10	*Burkholderiaceae_ unclassified*	7	*Xanthobacteraceae_ unclassified*	4
9	*Pseudomonas*	4	*Caulobacteraceae_ unclassified*	10	*Spirosoma*	7	*Aminobacter*	4
10	*Rhodanobacter*	4	*Hydrogenophaga*	10	*Pseudomonas*	6	*Burkholderia-Caballeronia-Paraburkholderia*	4

Table S12. The top 10 hubs ordered by Kleinberg's hub centrality score in the lettuce root-associated bacterial community networks grouped per urine-derived fertilizer treatment (ED: electrodialysis).

	**NPK Control**		**ED Concentrate**		**Hydrolyzed Urine**		**K-Struvite**	
#	**Genus**	**Hub Score**	**Genus**	**Hub Score**	**Genus**	**Hub Score**	**Genus**	**Hub Score**
1	*Rhodanobacteraceae_ unclassified*	1	*Sphingomonadaceae_ unclassified*	1	*JGI_0000069-P22_ge*	1	*Enterobacteriaceae_ unclassified*	1
2	*Mycobacterium*	0.81	*Massilia*	0.98	*Spirosoma*	0.88	*Pseudomonas*	0.83
3	*Cupriavidus*	0.81	*Nubsella*	0.96	*Mucilaginibacter*	0.69	*Burkholderia-Caballeronia-Paraburkholderia*	0.74
4	*Allorhizobium-Neorhizobium-Pararhizobium-Rhizobium*	0.67	*Rhizobiaceae_ unclassified*	0.95	*Gemmata*	0.65	*Pseudomonas*	0.63
5	*Allorhizobium-Neorhizobium-Pararhizobium-Rhizobium*	0.45	*Chlamydiales_ unclassified*	0.91	*Massilia*	0.61	*Xanthobacteraceae_ unclassified*	0.56
6	*Pandoraea*	0.37	*Azospirillum*	0.89	*Paludibaculum*	0.60	*Pseudomonas*	0.54
7	*Luteibacter*	0.33	*NS9_marine_group_ge*	0.87	*Burkholderiaceae_ unclassified*	0.60	*Enterobacteriaceae_ unclassified*	0.46
8	*Herbaspirillum*	0.32	*Methylophilus*	0.87	*Enterobacteriaceae_ unclassified*	0.59	*Sphingomonas*	0.42
9	*Leifsonia*	0.28	*Hydrogenophaga*	0.87	*Stenotrophomonas*	0.59	*Herbaspirillum*	0.37
10	*Burkholderia-Caballeronia-Paraburkholderia*	0.27	*Candidatus_ Protochlamydia*	0.86	*Bdellovibrio*	0.58	*Burkholderiaceae_ unclassified*	0.32

Table S13. The top 10 genera ordered by node degree in the lettuce root-associated bacterial community networks grouped per root zone. The degree of a node is the number of adjacent edges.

#	Rhizosphere		Rhizoplane		Rhizoplane + Endosphere	
	**Genus**	**Degree**	**Genus**	**Degree**	**Genus**	**Degree**
1	*Fimbriimonadaceae_ unclassified*	11	*Pandoraea*	9	*Pseudomonas*	8
2	*Burkholderia-Caballeronia-Paraburkholderia*	10	*Burkholderia-Caballeronia-Paraburkholderia*	9	*Burkholderia-Caballeronia-Paraburkholderia*	8
3	*Reyranella*	10	*Hyphomicrobium*	9	*Massilia*	8
4	*Hirschia*	10	*Sphingobium*	9	*Allorhizobium-Neorhizobium-Pararhizobium-Rhizobium*	7
5	*Pseudomonas*	9	*Enterobacteriaceae_ unclassified*	8	*Burkholderia-Caballeronia-Paraburkholderia*	7
6	*Enterobacteriaceae_ unclassified*	9	*Enterobacteriaceae_ unclassified*	8	*Herbaspirillum*	7
7	*Burkholderia-Caballeronia-Paraburkholderia*	8	*Enterobacteriaceae_ unclassified*	8	*Pelomonas*	7
8	*Burkholderia-Caballeronia-Paraburkholderia*	8	*Burkholderia-Caballeronia-Paraburkholderia*	7	*Reyranella*	7
9	*Luteibacter*	8	*Burkholderia-Caballeronia-Paraburkholderia*	7	*Devosia*	7
10	*Aminobacter*	8	*Rhodanobacter*	7	*Sphingomonas*	7

Table S14. The top 10 hubs ordered by Kleinberg's hub centrality score in the lettuce root-associated bacterial community networks grouped per root zone.

#	Rhizosphere		Rhizoplane		Rhizoplane + Endosphere	
	**Genus**	**Hub Score**	**Genus**	**Hub Score**	**Genus**	**Hub Score**
1	*Reyranella*	1	*Hyphomicrobium*	1	*Allorhizobium-Neorhizobium-Pararhizobium-Rhizobium*	1
2	*Fimbriimonadaceae_unclassified*	0.80	*Burkholderia-Caballeronia-Para-burkholderia*	0.96	*Burkholderia-Caballeronia-Paraburkholderia*	0.95
3	*Burkholderia-Caballeronia-Para-burkholderia*	0.74	*Burkholderia-Caballeronia-Para-burkholderia*	0.74	*Pelomonas*	0.90
4	*Hirschia*	0.70	*Rhodanobacter*	0.74	*Sphingomonas*	0.86
5	*Burkholderia-Caballeronia-Para-burkholderia*	0.68	*Devosia*	0.73	*Burkholderia-Caballeronia-Paraburkholderia*	0.75
6	*Aminobacter*	0.66	*Sphingobium*	0.72	*Fimbriimonadaceae_unclassified*	0.74
7	*Enterobacteriaceae_unclassified*	0.62	*Dyadobacter*	0.68	*Sphingopyxis*	0.63
8	*Burkholderia-Caballeronia-Para-burkholderia*	0.57	*Luteibacter*	0.67	*Sphingobium*	0.53
9	*Xanthobacteraceae_unclassified*	0.56	*Delftia*	0.61	*Alkanibacter*	0.50
10	*Spirosoma*	0.53	*Sphingomonas*	0.58	*Devosia*	0.47

Table S16. Overview of the habitats and characteristics of the key genera and families in the lettuce root-associated bacterial communities fertilized with urine-derived fertilizer (ED: electrodialysis).

Urine-Derived Fertilizer	Key Genera or Families	Habitats	Characteristics	References
ED Concentrate	*Cupriavidus*	Detected in the rhizosphere of plants in alkaline soil.	• N-fixing root nodule formation.<br>• *Cupriavidus* is closely related to the *Ralstonia* genus, whose members are soil-borne pathogens causing bacterial wilt.<br>• Increase shoot and root dry weight.<br>• Phosphate solubilization.	[78,116-120]
	Caulobacteraceae	Detected in the rhizosphere.	• Contains plant growth-promoting and inhibiting members.	[121]
	Kaistia	Detected in the tomato rhizosphere in alkaline soil.	• Plant growth-promoting or inhibiting effects are not known.	[122]
	Devosia	Detected in the rhizosphere of an aquatic legume.	• N-fixing root nodule formation.	[123]
	Gemmatimonas	Detected in rice paddies and the rhizosphere of artichoke in saline soil.	• Play a role in organic matter decomposition.	[124-126]
	Leptospira	Detected in inundated agricultural fields.	• Members that cause zoonotic disease in humans exist. Infection commonly occurs through contact via soil or water contaminated with urine from an infected animal.	[127]
	Aminobacter	Detected in the rhizosphere.	• Root nodule formation.<br>• Can use ammonia, nitrate, or urea as nitrogen sources.	[88,89]
	SH-PL14	Detected in the rhizosphere.	• *SH-PL14* is a member of the *Planctomycetales,* which is characterized by anaerobic oxidation of ammonium to N_2 (anammox).	[90,91]

Sphingomonas	Detected in soil, aquatic, and saline environments, and the rhizosphere.	• Plant growth-promotion.<br>• N-fixation.<br>• Phytohormone production.	[128]
Arcicella	Detected in wetland and aquatic environments.	• Plant growth-promoting or inhibiting effects are not known.<br>• Can use nitrate and ammonium as nitrogen sources.	[129,130]
Flectobacillus	Detected in rice paddy rhizosphere soil.	• *Flectobacillus* is closely related to the *Arcicella* genus.<br>• Can solubilize phosphate and potassium.	[92-94]
Pseudoxanthomonas	Detected in the rhizosphere and riverside soil.	• Reduce nitrite but not nitrate to N_2O.<br>• Show nematostatic activity.	[131-134]
Aeromonas	Detected in aquatic environments and halophyte rhizospheres.	• Phytohormone production.<br>• Contains human pathogenic members.	[135-137]
Acidovorax	Detected in lettuce rhizosphere.	• Contains plant pathogenic members that cause bacterial leaf spots and bacterial fruit blotch.	[85-87]
Pedobacter	Detected in the rhizosphere and aquatic environments.	• Nitrifying members exist.	[138-140]
Nubsella	Detected in an aquatic environment.	• *Nubsella* is closely related to the *Pedobacter* genus.	[141]
Flavobacteriales bacterium *NS9 marine group*	Detected in seawater.	• Plant growth-promoting or inhibiting effects are not known.	[142,143]
Methylophilus	Detected in the rhizosphere of hydroponically grown lettuce.	• Phosphate solubilization.<br>• Phytohormone production.<br>• Siderophore production.	[144,145]
Fimbriimonadaceae	Detected in ginseng field soil.	• Plant growth-promoting or inhibiting effects are not known.	[146]

Hydrolyzed Urine	*Gracilibacteria* bacterium *JGI 0000069-P22*	JGI 0000069-P22 has been detected in seawater. *Gracilibacteria* has been detected in rhizosphere soil.	• Plant growth-promoting or inhibiting effects are not known.	[147,148]
	Holosporaceae	Detected aquatic environments and in the root endosphere of Miscanthus.	• Can infect the unicellular ciliate *Paramecium*.<br>• Plant growth-promoting or inhibiting effects are not known.	[149,150]
	Zoogloea	Detected in forest soil and rice paddy soil.	• N-fixation.	[151,152]
	Eoetvoesia	Not yet detected in the root environment.	• Plant growth-promoting or inhibiting effects are not known.	[153]
	Edaphobacter	Detected in forest soils and wheat rhizosphere.	• Plant growth-promoting or inhibiting effects are not known.	[154,155]
	FBP_ge	Detected in coastal-steppe soils.	• Plant growth-promoting or inhibiting effects are not known.	[156]
	Acetobacter-aceae	Detected in the rhizosphere.	• Salt tolerant members exist.<br>• N-fixing and nodule forming.<br>• Plant growth-promotion.<br>• Phosphate solubilization.<br>• Phytohormone production.	[157-159]
	Massilia	Detected in the rhizosphere.	• Positively linked in abundance to high levels of *Rhizoctonia solani*.<br>• Antagonism towards *Phytophthora infestans*.<br>• Phytohormone production.	[105-108,160]
	Ignavibacteria bacterium *OPB56*	Detected in the rhizosphere of hydroponically grown durum wheat.	• Plant growth-promoting or inhibiting effects are not known.	[161]
	Sphingobium	Detected in the rhizosphere of lettuce.	• Can cause corky root of lettuce and is induced by free ammonia.	[102-104, 162,163]

			• Can degrade allelopathic pterostilbene, produced by plants to inhibit fungal infection, limiting its effectiveness.	
	Dyadobacter	Detected in the rhizosphere and aquatic environments.	• Nitrate reduction.<br>• Linked to Fusarium wilt and rice blast disease suppression.	[109,110,164]
	Reyranella	Detected in the rhizosphere of lettuce, in agricultural, forest, and desert soils, and a eutrophic lake.	• May promote the outbreak of bacterial wilt disease (*Ralstonia solanacearum*).<br>• Nitrate reduction.	[99-101,165-167]
K-Struvite	*Rhodanobacteraceae*	Detected in the rhizosphere and nitrate-rich subsurface environments (e.g., aquifers). Endophyte in tomato roots.	• Antagonism towards the root fungal pathogen *Fusarium solani*.<br>• Antagonism towards bacterial wilt disease (*Ralstonia solanacearum*).<br>• Promote plant growth under salt-stressed conditions.<br>• Produces antioxidant enzymes.<br>• Capable of complete denitrification.	[111-113]
	Enterobacteriaceae	Detected in the rhizosphere.	• Promote plant growth.<br>• Antagonism towards the root fungal pathogen *Fusarium solani*.<br>• Phytohormone production.<br>• N-fixation.<br>• Phosphate solubilization.	[78,114,115]
	Thiomonas	Detected in the rhizosphere and arsenic-contaminated environments.	• Can oxidize sulfur, iron, and arsenic.<br>• Use thiosulfate as an energy source.	[168-170]
